# Structural elucidation of the hexameric MmpS4-MmpL4 complex from *Mycobacterium tuberculosis*

**DOI:** 10.64898/2026.01.07.698164

**Published:** 2026-01-07

**Authors:** Jennifer C. Earp, Nicolas P. Lichti, Alisa A. Garaeva, Virginia Meikle, Michael Niederweis, Markus A. Seeger

**Affiliations:** 1 Institute of Medical Microbiology, University of Zurich, Zurich, Switzerland; 2 Present address: leadXpro, Villigen, Switzerland; 3 Department of Microbiology, University of Alabama at Birmingham, Birmingham, AL, USA

## Abstract

*Mycobacterium tuberculosis* contains thirteen Mycobacterial membrane protein Large (MmpL) transporters, which belong to the family of secondary active RND transporters. MmpL4 and MmpL5, together with their operon partners MmpS4 and MmpS5, export the mycobacterial siderophore mycobactin and the last resort TB drug bedaquiline. Recently, we determined a structure of the MmpL4 monomer in complex with desferrated mycobactin, which lacked a functionally essential coiled-coil domain predicted to extend far into the periplasm. Here, we present a cryo-EM structure of the hexameric (MmpS4)_3_-(MmpL4)_3_ complex, which was enabled by rational disulfide cross-links based on AlphaFold predictions. We observed density for the coiled-coil domain, which protrudes into the periplasmic space at an angle of around 60° relative to the symmetry axis of the MmpL4 trimer. In the context of the hexameric complex, MmpL4’s conformation differs strikingly from the one observed for monomeric MmpL4, which includes formation of a large cavity in the periplasmic domain and rearrangements of conserved proton coupling residues at the transmembrane domain. Our work provides an experimental workflow to obtain single particle cryo-EM structures of labile multiprotein complexes by AlphaFold-informed stabilization of predicted protein interfaces.

## INTRODUCTION

*Mycobacterium tuberculosis* encodes 13 resistance-nodulation-cell division (RND) transporters, referred to as the Mycobacterial membrane protein Large (MmpL) family [1]. Multiple MmpL transporters are of interest as novel targets for anti-TB drugs. This includes the only essential member of the MmpL family, the trehalose monomycolate (TMM) exporter MmpL3 [2, 3]. Extensive characterization by X-ray crystallography and cryo-EM revealed the molecular details of how MmpL3 inhibitors and TMM substrates are bound [4-6] and clinical trials for small molecules targeting MmpL3 are ongoing [7]. Two additional MmpL transporters, the siderophore (mycobactin) exporters MmpL4 and MmpL5, are also of clinical interest. These transporters are essential under low iron conditions and strongly contribute to virulence in mice [8, 9]. Importantly, MmpL4 and in particular MmpL5 confer resistance to bedaquiline, an antibiotic crucial for the treatment of multidrug resistant *M. tuberculosis* [10-13].

Like other RND transporters, MmpL proteins are proton-driven antiporters composed of a transmembrane domain and a periplasmic domain [2, 3]. The transmembrane domain consists of two pseudosymmetric six-helix bundles and contains conserved residues that are crucial for proton translocation. In case of MmpL3, MmpL4 and MmpL5, two DY-pairs located at the center of the transmembrane domain have been suggested to enable proton coupling [14-16]. The periplasmic domain is made up of two porter domains, referred to as the N-terminal (PN) and and C-terminal (PC) porter domain. These two domains are expected to undergo rigid body movements that allow substrate transport through these periplasmic domains similar to the conformational changes observed in the *E. coli* multidrug efflux pump AcrB [17]. Structures of MmpL3 in which the PN and PC adopt an open conformation enclosing a large cavity and of MmpL4 in which the domains adopt a closed conformation provided initial support for this hypothesis [4, 15]. However, the mechanistic basis of how these conformational changes are coupled to protonation of the DY-pairs in the transmembrane domain remains elusive.

Many but not all MmpL proteins contain an additional coiled-coil domain (CCD) inserted in the PC, a structural element extending far into the periplasm. The CCD was first described based on the AlphaFold prediction of MmpL4, and was proposed to be involved in multimerization of MmpL proteins [15]. Based on the presence or absence of the CCD the MmpL family can be divided into two phylogenetic cluster. The CCD is the hallmark of cluster I MmpL transporters which contains most members of the family and differentiates it from cluster II MmpL transporters that lack this domain (MmpL3, MmpL11 and MmpL13). Additionally, only cluster I MmpLs are associated with a periplasmic accessory protein called Mycobacterial membrane protein small (MmpS), which is composed of a single transmembrane helix and a periplasmic β-sheet sandwich domain. The genes encoding five out of the ten cluster I MmpL transporters (*mmpL1*, *mmpL2, mmpL4, mmpL5, mmpL6*) form an operon with genes encoding their respective MmpS proteins.

Despite the biological and clinical importance of cluster I MmpL transporters, including MmpL4 and MmpL5, the first structures were determined only recently. *M. smegmatis* MSMEG_1382 was the first published structure of a cluster I MmpL transporter, but the CCD was not resolved due to the flexibility of the domain [18]. The second published cryo-EM structure was that of monomeric *M. tuberculosis* MmpL4 in complex with its substrate mycobactin, determined by our lab [15]. To obtain this structure the CCD had to be genetically truncated as full-length MmpL4 preparations consisted of multimeric assemblies that were not amenable for structure determination [15]. Using truncated constructs of MmpL4 and MmpL5 we also demonstrated that this domain is essential for mycobactin and bedaquiline export in *M. tuberculosis* using a mycobactin toxicity assay and bedaquiline susceptibility assay [15].

A role of the CCD in trimer formation was uncovered in a biochemical study of the cluster I *M. smegmatis* MmpL10 transporter (MSMEG_0410) [19]. Additionally, a contribution of the MmpS protein to MmpL trimer formation was described based on single-molecule TIRF microscopy measurements of GFP-tagged MmpL5 in mycobacterial cells [20]. Recent structures of hexameric *M. tuberculosis* MmpS5-MmpL5 and *M. smegmatis* MSMEG_1381-MSMEG_1382 complexes confirmed that both the CCD and MmpS protein contribute to MmpL trimerization [16, 21]. As predicted by AlphaFold, three CCDs interact extensively to form an elongated stalk. However, apart from these interactions few other contacts are made directly between the MmpL protomers. Instead, the MmpS proteins form extensive contacts with the transmembrane and porter domains of multiple MmpL protomers and the MmpS β-sheet sandwich domains surround the CCD stalk [16, 22]. Even though both the CCD and MmpS contribute to MmpL trimerization, neither are strictly required to obtain a stable multimeric assembly of the MmpL complex. This was demonstrated by a structure of hexameric *M. tuberculosis* MmpS5-MmpL5 wherein the CCD was genetically deleted [22] and purification of trimers of full-length MmpL5 in the absence of MmpS5 [16].

MmpL4, MmpL5 and MSMEG_1382 co-purify with abundant acyl-carrier proteins (ACPs) from the respective expression host, namely *E. coli* ACP in case of MmpL4 [15] and AcpM in case of MmpL5 [16] and MSMEG_1382 [23]. Biochemical analysis showed that MmpL4 also interacts with MbtL (Rv1344), an ACP involved in mycobactin biosynthesis, suggesting that mycobactin biosynthesis and export are coupled processes [15].

In this work, we utilized the AlphaFold2 model of (MmpS4)_3_-(MmpL4)_3_ to rationally design disulfide bonds between MmpS4 and MmpL4. These engineered disulfide cross-links stabilized the MmpS4-MmpL4 interface, thereby enabling cryo-EM structure determination of hexameric MmpS4-MmpL4. This structure revealed a binding cavity in the periplasmic domain of MmpL4 and a tilted conformation of the CCDs.

## RESULTS

### Prediction of a hexameric MmpS4-MmpL4 complex using AlphaFold2

In order to purify MmpL4 from *E. coli, mmpL4* had to be expressed together with its operon partner *mmpS4* [15]. When full-length MmpL4 (including its CCD) was purified via a C-terminal GFP-tag, the sample predominantly eluted as a broad peak from the size exclusion chromatography (SEC) column, corresponding to MmpL4 multimers of varying stoichiometries [15]. A later-eluting peak contained the MmpL4 monomer and suffered from protein degradation. SDS-PAGE analysis and mass-spectrometry analyses of the early-eluting peak revealed that MmpS4 was co-purified, as well as the acyl-carrier protein ACP of the expression host *E. coli*. However, when attempting to determine a cryo-EM structure of the multimeric MmpS4-MmpL4 complex, the sample quality was poor, only permitting reconstruction of a 6.3 Å map of the MmpL4 monomer in complex with ACP [15]. Hence, in our hands, the detergent-purified MmpS4-MmpL4 complex was not stable enough to be structurally investigated.

**Figure 1 ∣:**
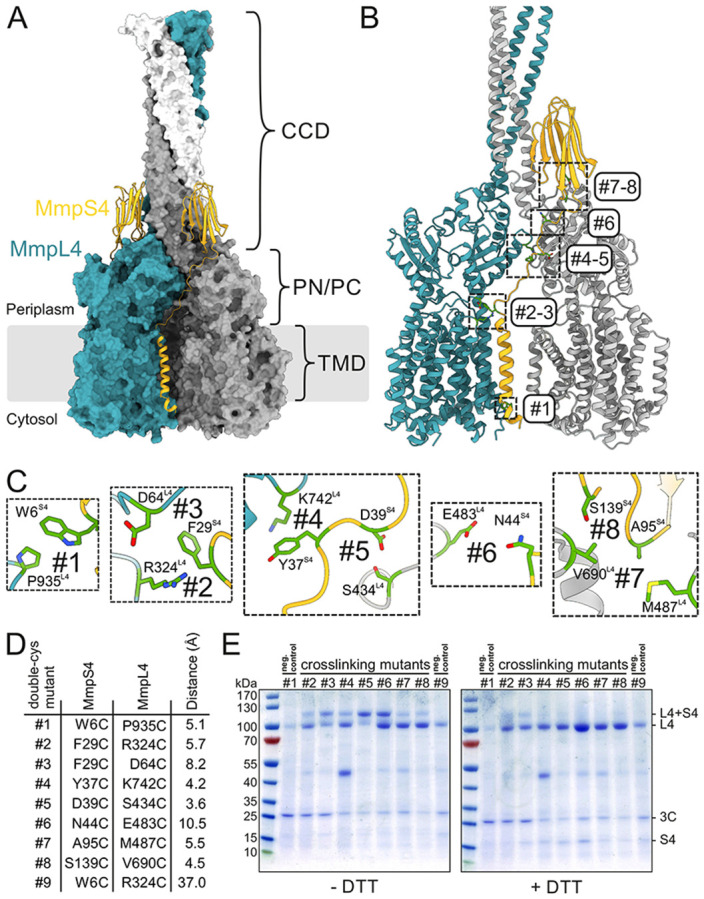
Engineered MmpS4-MmpL4 disulfide cross-links based on AlphaFold2 prediction. (**A**) AlphaFold2 prediction of the hexameric (MmpS4)_3_-(MmpL4)_3_ complex. The three protomers of MmpL4 are shown in surface presentation and colored petrol, grey and white. The MmpS4 protomers are shown as yellow cartoon. The boundary of the cytoplasmic membrane is indicated by grey lines. The locations of the transmembrane domains (TMD) and the periplasmic PN/PC domains are indicated. MmpL4 is predicted to form a large coiled-coil domain (CCD) extending into the periplasm. (**B** and **C**) Based on the AlphaFold2 model, eight double-cysteine mutants (#1-#8) were generated, which exhibit a close distance between MmpL4 and MmpS4. The original residues that were substituted by cysteines are shown as sticks. The mutants #2-#8 are expected to form spontaneous disulfide cross-links in the oxidative environment of the periplasm. (**D**) List of characterized double cysteine mutants. Whenever possible, the distances were measured between the γ-atoms of the respective residues of MmpS4 and MmpL4. In case of alanine and proline residues, the atom nearest to the γ-atom of the partner residue was used to measure the distance. (**E**) Non-reducing (left) or reducing (right) SDS-PAGE analysis of purified cysteine cross-linking mutants of MmpS4-MmpL4, stained by coomassie. The negative control cross-linking pair #1 was not expected to form a disulfide bond because it is located in the reducing environment of the cytosol. The negative control cross-linking pair #9 bears cysteines that are too far apart to form a disulfide bond.

Therefore, we used AlphaFold2 to predict the multimeric complex including three MmpL4 and three MmpS4 chains [24]. AlphaFold2 generated a model with high values for the predicted local distance difference test (pLDDT) score and importantly also for the predicted alignment error (PAE) over the entire polypeptide chains of MmpL4 and MmpS4 indicating a high confidence in the predicted interactions (Fig. 1A, Fig. S1). The most characteristic hallmark of the (MmpS4)_3_-(MmpL4)_3_ model is the CCD of MmpL4 which extends along the three-fold symmetry axis 130 Å far into the periplasm. MmpS4 is predicted to be anchored in the cytoplasmic membrane via an N-terminal single transmembrane helix. If we follow the polypeptide sequence of one MmpS4 protomer in the AlphaFold2 model, the transmembrane helix of MmpS4 associates with the 12-transmembrane helix bundle of the first MmpL4 protomer (petrol in Fig. 1A) by interacting with transmembrane helix 8. A linker devoid of secondary structures bridges the crevice formed between the first (petrol) and second (white) MmpL4 protomer, thereby establishing interactions with both MmpL4 protomers. The C-terminal domain of MmpS4 features the topology of a β-sheet sandwich [8, 25], wherein two β-sheets each comprising four β-strands each stack against each other. This architecture is further reinforced by a disulfide bond connecting that connects the sheets. The MmpS4 β-sheet sandwich establishes extensive contacts with the CCD of MmpL4, especially the α-helical hairpins donated from the second (white) and the third (grey) MmpL4 protomer. In summary, MmpS4 is predicted to establish extensive contacts with all three MmpL4 protomers, thereby stabilizing the MmpL4 trimer, and in particular the CCD.

### Experimental validation of the AlphaFold2-predicted MmpS4-MmpL4 complex

We used targeted disulfide cross-linking to experimentally validate the AlphaFold2-predicted MmpS4-MmpL4 complex. Spontaneous disulfide bonds are expected to form in the oxidative environment of the periplasm of *E. coli* if cysteine pairs are sufficiently close to each other, especially if they are placed in flexible loop regions. The same experimental strategy was previously utilized to study the conformational cycling of the RND transporter AcrB [26], but also more recently to validate the MmpS5-MmpL5 complex structure [16]. To avoid unspecific reactions with free thiol groups, we mutated five out of the seven naturally occurring cysteines of MmpL4 into serines (see methods). We decided to keep two highly conserved cysteine-pairs that form disulfide bonds, the first one at the tip of the CCD of MmpL4 and the second one between the β-sheets of MmpS4. The resulting cys-depleted MmpS4-MmpL4 construct behaved similarly to the original progenitor in terms of protein yield and quality (Fig. S2). In addition, cys-depleted MmpS4-MmpL4 was fully functional in our mycobactin toxicity assay (Fig. S3).

Based on the AlphaFold2 prediction we introduced eight cysteine pairs (numbered #1- #8) with close distances between the thiol group into flexible loops along the MmpS4-MmpL4 interface (Fig. 1B and C). Cysteine pair #1 located at the cytosol served as a negative control, because it is not expected to form spontaneous disulfide bonds due to the reducing environment. Cysteine pair #9 was included as an additional negative control, because its thiols are 37 Å away from each other and therefore cannot form disulfide bonds (Fig. 1D). The respective proteins were produced in *E. coli*. Prior to the detergent solubilization step, n-ethylmaleimide was added to react with free reactive thiols and prevent undesired disulfide cross-links between transporter complexes during detergent extraction and subsequent purification steps. Subsequently, the transporter complexes were purified via GFP attached C-terminally to MmpL4 and analyzed by non-reducing and reducing SDS-PAGE (Fig. 1E). Cysteine pairs #2-8 established variable degrees of MmpS4-MmpL4 cross-linking. As expected, the cytoplasmic pair #1 and the negative control pair #9 did not cross-link, thereby validating the experimental approach. The degree of cross-linked protein for cysteine pair #5 (D39C^MmpS4^-S434C^MmpL4^), which according to the AlphaFold2 prediction has the closest distance of 3.6 Å between the thiol moieties, was the highest. In summary, the engineered cysteine cross-links provided experimental evidence for the interaction between and trimerization of MmpS4 and MmpL4 as predicted by the AlphaFold2 model.

### Cryo-EM structure of the (MmpS4)_3_-(MmpL4)_3_ complex

**Figure 2 ∣:**
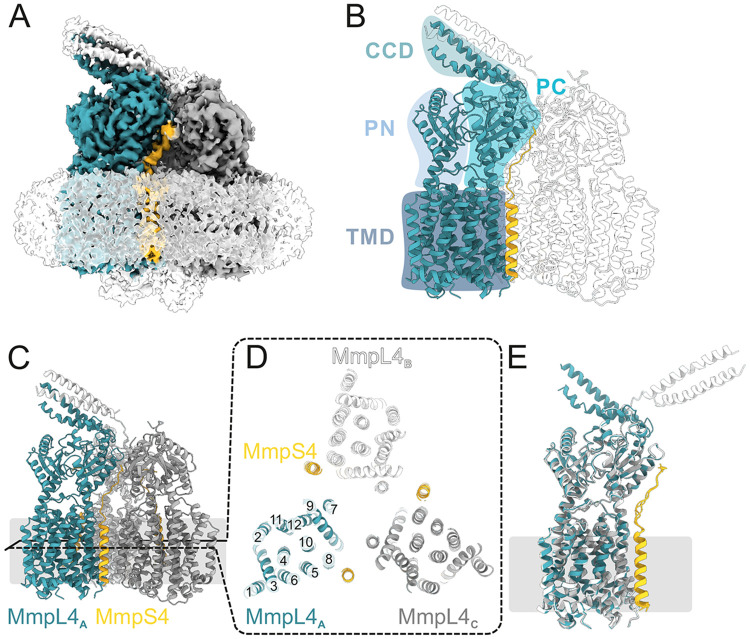
Structure of the hexameric (MmpS4)_3_-(MmpL4)_3_ complex. (**A**) Left panel; Cryo-EM map of the hexameric (MmpS4)_3_-(MmpL4)_3_ complex processed using C1 symmetry viewed along the membrane plane and contoured at 4σ. The detergent micelle and weak density for *E. coli* ACP are shown as white, transparent density. MmpL4 protomers are colored in petrol, grey and white, MmpS4 in yellow. Density for a tilted CCD formed out of two α-helical hairpins donated from MmpL4 protomers A (petrol) and B (white) is visible. (**B**) Schematic labeling of MmpL4 domains. (**C**) Structural model of the asymmetric (MmpS4)_3_-(MmpL4)_3_ complex shown as cartoon shown along the membrane plane (left). (**D**) Transmembrane helices of MmpL4 and MmpS4 viewed from the periplasm. The transmembrane helices of MmpL4 are numbered. (**E**) Superimposed MmpS4-MmpL4 monomers of asymmetrically processed (MmpS4)_3_-(MmpL4)_3_ complex, showing the asymmetric orientation of the α-helical hairpins.

The cysteine cross-linking pair #5, which exhibited the strongest cross-linking efficiency, was purified (Fig. S2) and analyzed by single particle cryo-EM, resulting in a high-resolution reconstruction of the hexameric (MmpS4)_3_-(MmpL4)_3_ complex (Fig. 2, Fig. S4, Fig. S5, Table S1). We obtained a 3.1 Å map without applying symmetry during data processing (Fig. S4). An additional density for the *E. coli* ACP was present at the cytosolic interface of the transmembrane domain, similar to what has previously been described in the structure of the MmpL4 monomer. Because this density was only weakly defined (Fig. 2A, Fig. S4), we did not include it in our model. The C1 cryo-EM map contained well-defined density for the transmembrane helices and part of the flexible linkers of MmpS4, but not for its β-sheet sandwich domains (Fig. 2A, Fig. S5). Well-resolved density is present for the transmembrane domains and the periplasmic PN and PC domains of MmpL4, whereas the CCD is less well defined (Fig. 2A and B). Strikingly, the CCD is tilted by around 60 ° away from the threefold symmetry axis rather than extending along it as predicted by AlphaFold2. Only two out of three α-helical hairpins of the three MmpL4 protomers are resolved (Fig. 2C), which may explain its tilted conformation. Using a local refinement approach with a mask containing the CCD and the uppermost regions of MmpL4, the cryo-EM density of the CCD and the loops connecting to the PC domain of MmpL4 was further improved (Fig. S4 and Fig. S5), enabling us to build a model of the lower part of the CCD, including the linkers connecting to the PC of MmpL4. Model building revealed that the tilted CCD is composed of the α-helical hairpins of the polypeptide chains A and B. To assess whether there are asymmetries in other parts of the structure, we superimposed the individual protomers of the (MmpS4)_3_-(MmpL4)_3_ complex analyzed using C1 symmetry (Fig. 2E). Apart from the two resolved α-helical hairpins, the MmpL4 core domains and the resolved parts of MmpS4 differ by a RMSD of less than 0.6 Å and are therefore structurally identical considering the map resolution of around 3 Å. The fact that the core domains of MmpS4 and MmpL4 do not deviate between the protomers argues against the possibility that the cross-links established between them cause the (asymmetric) tilt of the CCD. To increase the resolution of the core parts of the (MmpS4)_3_-(MmpL4)_3_ complex, we applied C3 symmetry resulting in a map with a resolution of 2.9 Å (Fig. S4). Using this data, we built a model comprising residues E16-M487 and V690-R939 of MmpL4 and residues T5-T40 of MmpS4.

### Identification of a deep cavity accommodating a detergent molecule

**Figure 3 ∣:**
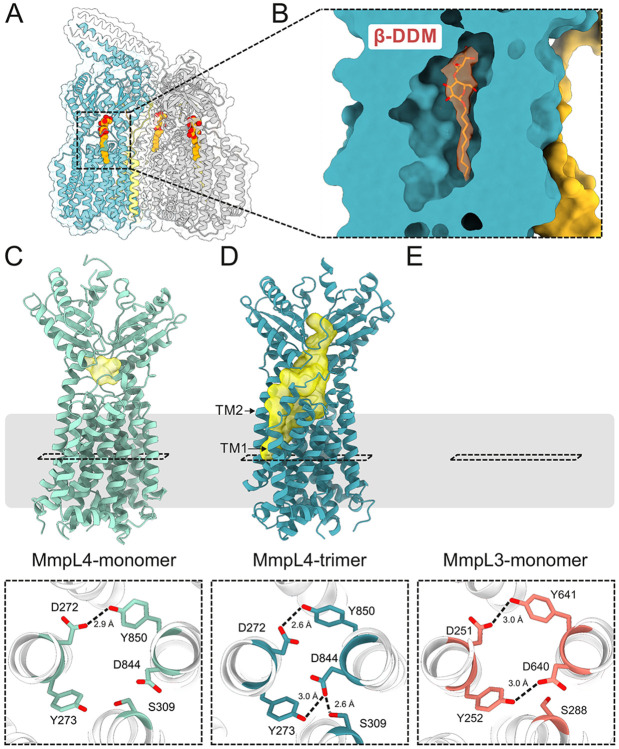
Detergent binding in a deep cavity extending into the periplasmic domain of MmpL4. (**A**) Structural model of hexameric (MmpS4)_3_-(MmpL4)_3_ complex processed using C1 symmetry. Three detergent molecules (β-DDM) bound to each of the MmpL4 protomers are depicted as red/orange spheres. (**B**) Enlarged view of the β-DDM binding site of chain A. Non-proteinaceaous density (contoured at 6.8σ) interpreted as β-DDM is depicted in orange. (**C**–**E**) Cavity analysis of monomeric MmpL4 (PDB ID: 9GI0) (**C**), trimeric MmpL4 (this work, PDB ID: 9SYJ) (**D**) and monomeric MmpL3 (PDB ID: 7NVH) (**E**) using the 3V tool. Cavities are shown in yellow and the membrane boundary is indicated. The bottom row shows the conserved DY-pairs and S309(MmpL4)/S288(MmpL3) viewed from the periplasm. Hydrogen bonds are indicated by dashed lines.

A systematic inspection of non-proteinaceous densities revealed a strong detergent-like density in a cavity formed between the PN and PC domains and the upper part of the TMD (Fig. 3A and B). We modeled the density as a N-dodecyl β-D-maltoside (β-DDM) molecule, the detergent used to extract and purify the transporter complex. The two sugar moieties are sandwiched between the PN and PC and the twelve carbon atoms of the aliphatic chain are accommodated in a hydrophobic patch in the transmembrane domain (Fig. 3B). We did not observe density for β-DDM at this location in the monomeric MmpL4 structure, although β-DDM was also used for protein extraction and purification. Using the 3 V tool [27], we analyzed the cavity that accommodates β-DDM in the (MmpS4)_3_-(MmpL4)_3_ complex and found that it spans from the TMD into the interface between PN and PC (Fig. 3D). The cavity does not open up to the periplasm as observed for the open conformation of MmpL3 (Fig. 3E). Instead, an entrance is found between transmembrane helices 1 and 2, at the same position at which Fountain et al. observed a prominent non-proteinaceous density in the MmpS5-MmpL5 complex structure [22]. In striking contrast, monomeric MmpL4 only contains a very small, occluded cavity at the same location (Fig. 3C). We also noted extra-densities wedged between the transmembrane helix bundles at the inner leaflet of the membrane, in analogy to the lipids observed in the MmpS5-MmpL5 structures that were suggested to stabilize the MmpL5 trimer [16, 22]. Because they were not sufficiently defined to unambiguously differentiate between lipid or detergent molecules we refrained from interpreting them. It should be noted that we purified the MmpS4-MmpL4 complex from *E. coli* as expression host, whereas the MmpLS5-MmpL5 complexes were both purified from *M. smegmatis*.

### Comparison of monomeric and trimeric MmpL4 structures

**Figure 4 ∣:**
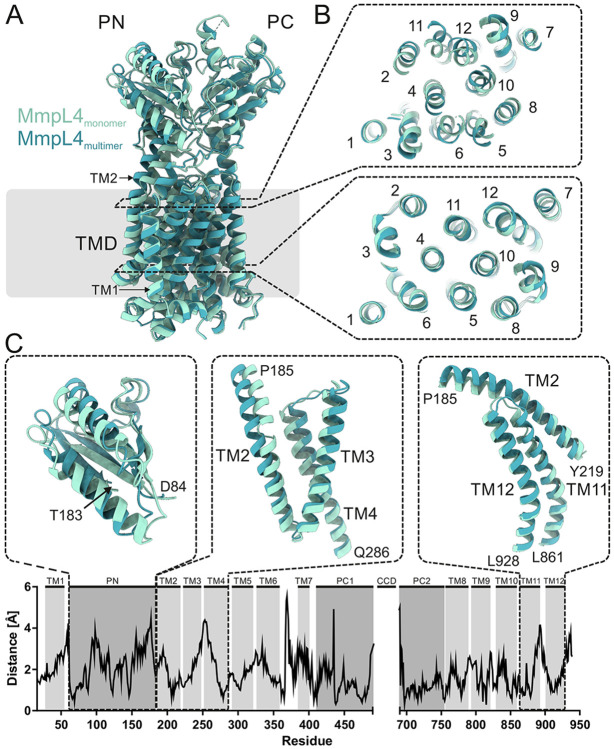
Systematic structural comparison of monomeric versus trimeric MmpL4. (**A** and **B**) Previously determined monomeric MmpL4 colored in light blue (PDB ID: 9GI0) was superimposed onto trimeric MmpL4 colored in dark blue (chain A of structure obtained using C1 symmetry, PDB ID: 9SYJ). (**B**) Two slices along the membrane plane are shown from the periplasm onto the TMDs at the two heights indicated by the dashed rectangles in (**A**). (**C**) C_α_-atom distance plot between monomeric and trimeric MmpL4. The respective TM helices and domains are indicated above. For three structural elements showing marked differences, the superimposed structures are shown as cartoon above the plot. No structural information was available for the CCD and the C-terminus. The overall RMSD is 2.14 Å over 722 residues.

To systematically analyze the conformational differences between monomeric and multimeric MmpL4, we used the Needleman–Wunsch algorithm implemented in ChimeraX to superimpose the 3.0 Å MmpL4 monomer structure (PDB ID: 9GI0) onto the first MmpL4 protomer (chain A) of the asymmetric (MmpS4)_3_-(MmpL4)_3_ complex structure determined at 3.1 Å (Fig. 4A). The RMSD was 2.14 Å over 722 residues, demonstrating that MmpL4 exhibits substantial structural differences depending on whether it is a monomeric protein or embedded in the hexameric complex. When plotting the C_α_-atom distance of monomeric versus trimeric MmpL4 (Fig. 4C), we found that conformational differences affect all parts of the transporter. Most prominently, we observed a rotation of the PN domain (Fig. 4C), which is responsible for creating the deep cavity accommodating β-DDM in the complex structure (Fig. 3A and B). Intriguingly, we also observed a rearrangement of the conserved DY-pairs, four key residues residing at the center of the transmembrane domain that are suggested to couple active transport to the proton-gradient [15] (Fig. 3C and D, Fig. S6). While Y273 and D844 are around 5 Å apart in monomeric MmpL4 (Fig. 3C), they engage in a hydrogen bond in the context of the hexameric complex (Fig. 3D) in a similar configuration as observed for MmpL3 (PDB ID: 7NVH) (Fig. 3E). These rearrangements may be coupled to protonation events at D844 in a similar manner as characterized for the proton translocating aspartates in the drug efflux pump AcrB [28]. Concomitant with the subtle rearrangements in the proton-coupling DY-pairs, the twelve transmembrane helices of MmpL4 change their position (RMSD of 1.78 Å over 369 residues) to a similar extent as the conformational changes observed for the TMD of asymmetric AcrB [29]. In MmpL4, the conformational changes mostly affect the upper parts of the transmembrane helices, in particular transmembrane helix 2-4 and 11-12 (Fig. 4B and C). These conformational changes result in a larger distance between transmembrane helices 1 and 2, creating the channel entry that leads to the periplasmic cavity (Fig. 3D). It remains to be shown in future work whether the structural differences observed between monomeric and multimeric MmpL4 represent alternate conformational states that are relevant for efflux function.

### Structural features of MmpL CCDs analyzed by AlphaFold

The presence of defined density for the tilted CCD consisting of α-helical hairpins of only two MmpL4 protomers suggests that it represents a thermodynamically stable intermediate. The density for the CCD was less well defined than other parts of MmpL4, suggesting that the CCD is rather flexible (Fig. S5). To gain further insights into this unique structural element, we used AlphaFold3 to predict the CCDs of all cluster I MmpL proteins of *M. tuberculosis* (Fig. S7A) and performed a sequence analysis of this region (Fig. S7B). In terms of phylogeny, Cluster I MmpL proteins can be further subdivided into two groups, the first one encompassing MmpL1, MmpL2, MmpL4, MmpL5, MmpL6 and MmpL9 (which apart from MmpL9 are encoded in an operon with a respective *mmpS* gene) and the second one encompassing MmpL8, MmpL10 and MmpL12 (Fig. S7). MmpL7 cannot be clearly assigned to either of the groups. The predicted CCDs differ quite strikingly between the two phylogenetic groups, with the second group (MmpL8, MmpL10 and MmpL12) being substantially longer and containing kinks and discontinuities in the α-helices. It should be noted that the AlphaFold3 pLDDT values were less confident for MmpL7, MmpL8, MmpL10 and MmpL12 as compared to the other MmpLs, and future experimental structures will be required to validate their correctness.

**Figure 5 ∣:**
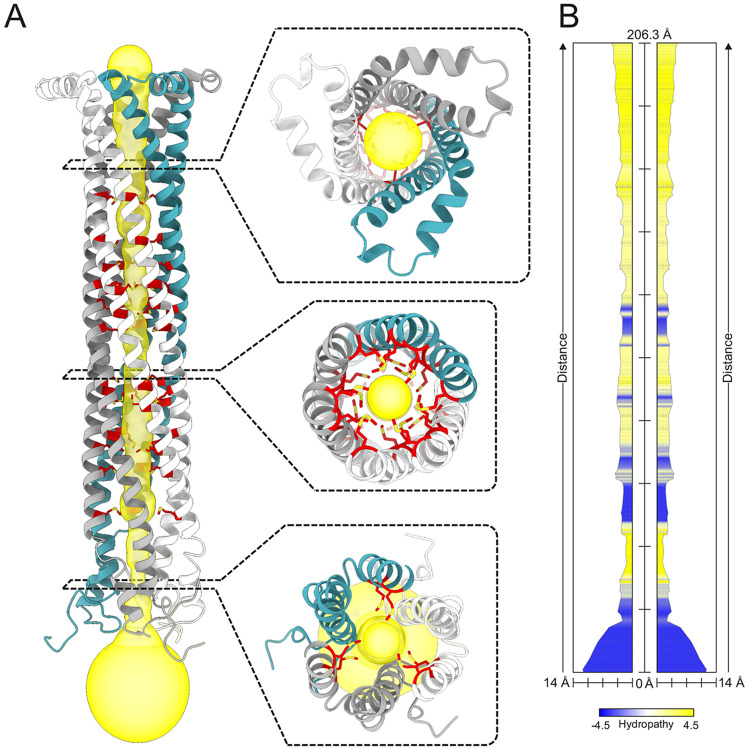
Channel prediction of MmpL4's CCD. (**A**) The CCD of trimeric MmpL4 as predicted by AlphaFold3 and shown as cartoon. A continuous channel leading through the CCD interior was calculated by the MOLEonline tool and shown as yellow surface. Methionine side chains lining the channel surface are shown as red sticks. Three top views along the pore are shown as insets. (**B**) Radius and biophysical properties of the channel shown in (**A**) calculated by the MOLEonline tool.

The most notable feature of the CCDs is the enrichment of methionine side chains pointing towards the enclosed channel for many, but not all MmpL proteins (Fig. 5A, Fig. S7). The channel wall of MmpL4’s CCD, for example, is lined with 45 methionines (15 per α-hairpin). By contrast, MmpL6 has one methionine per α-hairpin, suggesting that the methionines are not *per se* needed for the assembly of the CCD, but instead likely play a functional role during transport.

The CCD channel radius of all MmpL proteins appears to be very narrow. The MOLEonline tool [30] predicts several constrictions and a minimal channel radius of 1.1 Å for the CCD of MmpL4 (Fig. 5B). It should be kept in mind that these analyses were performed with AlphaFold predictions and not with experimental structures. In its proposed closed conformation, the channel is too narrow to let bulky substrates such as desferrated mycobactin or bedaquiline pass through, even if one considers flexibility of long methionine side chains. Further experimental studies will be required to deepen our mechanistic understanding of the CCD, in particular with regards to its role in substrate transport.

## DISCUSSION

Structure predictions using AlphaFold have become a standard step in every structural biology project, due to its ease of use and the high accuracy of its predictions [24]. Predictions of multi-protein complexes by AlphaFold2 multimer (and more recently AlphaFold3) are especially attractive, because they provide potential cues about the interaction interfaces as well as possible multimeric assemblies [31, 32]. In the context of MmpL and MmpS proteins, AlphaFold has been extensively used by us and others to firstly identify the CCD in MmpL4 and MmpL5 and to secondly predict its involvement in trimerization of the proteins [15, 16, 19, 22]. In our previous study on MmpL4, we cloned a transporter construct devoid of the predicted CCD to enable the determination of a highly resolved cryo-EM structure of the MmpL4 monomer [15]. An analogous strategy was also chosen by Fountain et al. to obtain the multimeric MmpS5-MmpL5 structure [22]. In this study, we used the hexameric structure of MmpS4-MmpL4 predicted by AlphaFold2 as the basis to introduce engineered cysteine cross-links. This strategy allowed us to biochemically validate the predicted model and facilitated its structural determination. The procedure described in this work may serve as a blueprint on how AlphaFold predictions can aid the rational stabilization of difficult-to-handle protein complexes. Other groups have determined cryo-EM complex structures of the MmpS4-MmpL4 homologues MmpS5-MmpL5 [16, 22] and MSEMG_1381-1382 [21]. These structures were determined without cross-linking, either by purifying the complexes from *M. smegmatis* [16, 22] (instead of *E. coli* as in this study) or by using grid preparation methods that avoid purification of the membrane protein complexes after solubilization [21]. In both approaches, hexameric complexes composed of three MmpS and three MmpL proteins were observed, demonstrating that cross-linking via engineered cysteines did not result in structural artefacts. In addition, the MmpS4_3_-MmpL4_3_ complex we obtained is structurally very similar to the MmpL5 structures obtained by the other two groups (RMSD values smaller than 1.4 Å for the core domains of MmpL4/MmpL5) (Fig. S8).

AlphaFold predictions and structures of the multimeric assembly of MmpS-MmpL proteins presented in this study and by others [16, 21, 22] provided important insights into the architecture of these unique efflux machineries. The structures established the role of the MmpS protein in stabilizing the MmpL trimer by interactions of every MmpS protomer with all three MmpL protomers. We did not observe density for the β-sheet sandwich domains of MmpS4, nor were the respective domains of MmpS5 observed in the symmetric hexameric MmpS5-MmpL5 complex [16]. Our cross-linking data, however, strongly suggests that the β-sheet sandwich domains of MmpS4 interact with the CCD in the configuration predicted by AlphaFold2 (see cross-links of cysteine pairs #7 and #8 in Fig. 2A) and this was confirmed by the cryo-EM structure of MSEMG_1381-1382 [21]. We therefore hypothesize that the role of the β-sheet sandwich domains of MmpS proteins is to further stabilize the fully assembled CCD and orchestrate the orientation of the CCD in the periplasm.

The CCDs of cluster I MmpL proteins are particularly interesting from a structural and functional point of view. In their fully assembled, upright and trimeric conformation, the CCDs protrude into the periplasm, as noted in our previous MmpL4 study [15] and also suggested by other research groups [16, 21, 22]. A length estimate based on the cryo-ET analysis of the mycobacterial cell membranes suggests that the upright CCD does not bridge the entire periplasmic space of *M. tuberculosis* [22], suggesting that further protein partners are needed to complete the bridge. In the hexameric (MmpS4)_3_-(MmpL4)_3_ complex structure presented in this work, the CCD adopts a partially assembled, tilted conformation, consisting of only two α-helical hairpins. A general finding from our and other studies is the overall flexibility of the CCD. In our structure, the density for the tilted CCD is less defined than other parts of the transporter (Fig. S5). Similarly, the CCD of MmpL5 was poorly defined in the MmpS5-MmpL5 complex structure determined with full-length MmpL5 [16], while in the study of Fountain et al., the CCD of MmpL5 was genetically truncated [22]. In contrast, the MSEMG_1381-1382 complex structure revealed a fully assembled, upright CCD and a clear density for the β-sheet sandwich domains of the MmpS protein intimately interacting with the CCDs, as predicted by AlphaFold2 [21], which may be explained by a more robust CCD of MSEMG_1382 compared to MmpL4 and MmpL5 or additional stabilization by the β-sheet sandwich domains of MSMEG_1381.

The channel radius of the MmpL4 CCD is very narrow (minimum of 1.1 Å) according to the AlphaFold3 predition (Fig. 5). In addition, the cryo-EM structure of MSMEG_1382 revealed a constriction in the CCD channel [21]. Even if the methionine side chains lining the channel wall are considered to be flexible, large conformational changes of the α-helical hairpins relative to each other would be required to allow passage of molecules as large as mycobactin and bedaquiline. In the context of the AcrAB-TolC efflux machinery, iris-like opening and closing movements have been described for the outer membrane component TolC [33], and similar helical twisting may widen the channel of the CCD. Alternatively, one might speculate that the substrates are transported along the CCD surface, potentially requiring further protein partners. It is interesting to note that siderophore secretion and drug efflux by MmpL4/MmpL5 systems involves at least two additional proteins, namely the periplasmic protein Rv0455, which was shown to be essential for mycobactin efflux [34] and an outer membrane channel, whose identity remain elusive. A complete molecular understanding of the transport mechanism through the MmpL4/MmpL5 systems hence requires the identification of the missing components and structural information about their interactions.

Finally, the route of substrate transport through the transmembrane domain and periplasmic domains of MmpL4 and MmpL5 remains to be further investigated. In our first study on MmpL4, we identified a mycobactin binding site that is accessible from the cytosol [15]. Fountain et al. recently performed extensive mutagenesis and gain-of-function experiments on MmpL5 and thereby identified several residues in the periplasmic domains involved in bedaquiline recognition and transport [22]. While the authors did not find a bedaquiline binding site, they identified a cleft between TM1 and TM2 which is larger in the multimeric assembly versus the MmpL5 monomer and contains non-proteinaceous density of unknown identity [22]. Similarly, in the context of the MmpS4-MmpL4 complex, we observed a large cavity occupied by β-DDM in the periplasmic domain, which has its entrance between TM1 and TM2. As has been learned for the multidrug efflux pump AcrAB-TolC through more than two decades of work, the identification of substrate transport routes is complex and not all substrates are transported through the same channels [35-37]. Interestingly, a subset of substrates including β-lactams, fusidic acid and β-DDM have been proposed to use a channel extending from a groove formed by TM1 and TM2 to reach their binding pocket [37]. A similar route may be taken by mycobatin and bedaquiline, though it has to be considered that AcrB contains a periplasmic domain architecture that is strikingly different from those of MmpL proteins.

Common to all transport routes through AcrAB-TolC is the necessity of the AcrB protomers (corresponding to MmpL4 and MmpL5 in our system) to undergo large conformational transitions resulting in the opening and closure of transport channels in the periplasmic domains to drive transport [17, 26, 38]. As a consequence, homotrimeric AcrB assumes an asymmetric conformation with pronounced structural deviations between the protomers, that reflect different protonation states of the key residues in the TMD responsible for coupling proton translocation to active transport [17, 28]. Apart from the tilted CCDs, the MmpL4 trimer in the multimeric structure presented in this work is highly symmetric (Fig. 4). Likewise, no notable asymmetries were reported for the MmpL5 and the MSEMG_1382 trimers [16, 21, 22]. In contrast, we noted pronounced conformational differences of MmpL4 in its monomeric versus its trimeric context which are accompanied by conformational changes to the proton-translocating DY-pair residues. Further studies will be required to fully uncover the conformational cycle of MmpL proteins in their multimeric assemblies, which will be crucial to understand the mechanism(s) by which these unique multi-subunit efflux systems export mycobactin as well as clinically important TB drugs.

## METHODS

### Strains, media and antibiotics

The *Escherichia coli* K-12 strain MC1061 was used for cloning and heterologous protein expression. Luria Broth (LB) liquid cultures and agar were used for cloning and Terrific broth (TB) liquid culture for protein expression. In *E. coli*, 100 μg ml^−1^ ampicillin and 200 μg ml^−1^ hygromycin were added to the medium when required. *M. tuberculosis* strains were grown at 37 °C in Middlebrook 7H9 liquid medium (Difco Laboratories) supplemented with 0.2% glycerol, 10% ADS, 0.2% casamino acids, 24 μg/ml pantothenate, or 0.02% tyloxapol and 20*u*M hemin. Hartmans de Bont (HdB) minimal medium (500 μM MgCl_2_, 7 μM CaCl_2_, 1 μM NaMoO_4_, 2 μM CoCl_2_, 6 μM MnCl_2_, 7 μM ZnSO_4_, 1 μM CuSO_4_, 15 mM (NH_4_)_2_SO_4_, 12 mM KH_2_PO_4_, pH 6.8) containing 1% (v/v) glycerol was supplemented with 0.2% casamino acids, 24 μg/ml pantothenate. 0.02% tyloxapol and 20 μM hemin and was used for the Alamar Blue assays. Hygromycin was added at a concentration of 50 μg ml^−1^ if needed.

### Model predictions using AlphaFold2 and AlphaFold3 and channel predictions

The structure of the MmpS4-MmpL4 trimeric complex was predicted using AlphaFold2 (Parafold setup) in multimer mode [24, 39]. The structures of CCDs of MmpL proteins were predicted using the web interface of AlphaFold3 [40] by first predicting the respective hexameric MmpS-MmpL (in case a MmpS protein is part of the complex) or trimeric MmpL complexes. For simplicity, we only show the CCDs of these structures. The channel properties of the predicted CCD of MmpL4 were analyzed using the MOLEonline tool [30].

### Cloning of mmpS4-mmpL4 cross-linking variants

The gene for *mmpS4*-*mmpL4-cys-depleted* (wherein C39, C319, C335, C393 and C780 of MmpL4 are substituted by serines) was synthesized by Twist Biosciences into the fragment exchange (FX) cloning compatible entry vector pINIT (Addgene #46858) [41]. The last 123 nucleotides of *mmpL4* were codon optimized for synthesis due to their high GC content. The full nucleotide sequence of cys-depleted *mmpS4/mmpL4* is shown in Table S3. The *mmpS4-mmpL4-cys-depleted* gene was subcloned into the expression vector pBXC3GH (Addgene #47070) by FX cloning [42]. Nine cysteine pairs were introduced into pBXC3GH_*mmpS4-mmpL4_cys-depleted* following the QuikChange site-directed mutagenesis protocol using the primers listed in Table S2. For example, for the W6C^MmpS4^-P935C^MmpL4^ cysteine pair first the W6C^MmpS4^ mutation was introduced to *mmpS4* using the primers mmpS4_W6_Fw and mmpS4_W6_Rv. After confirmation by Sanger sequencing, the P935C^MmpL4^ mutation was introduced into *mmpL4* using the primers mmpL4_P935_Fw and mmpL_P935_Rv. The final constructs were validated by Oxford Nanopore sequencing. A 3xFLAG tag was introduced to the pML4324 vector by Gibson assembly using primers FLAG_fw and FLAG_rv for detection of protein production levels by Western Blotting. Then the *mmpS4/mmpL4-cys-depleted* was subcloned into the pML4324_3xFLAG vector by Gibson assembly.

### Cross-linked MmpS4-MmpL4 purification

The MmpS4-MmpL4 cysteine cross-linking constructs were expressed in *E. coli* MC1061. Culture volumes were either 0.6 L TB supplemented with 100 μg/ml ampicillin for small scale purification to determine crosslinking efficiency or 3 L medium to purify sufficient material for cryo-EM analyses. Cells were incubated at 37 °C for 2 h and 25 °C for 40 minutes after which protein expression was induced with 0.02% L-arabinose. Cells were harvested after 16 hours by centrifugation for 15 minutes at 4 °C and 8,000 g in a F9-6x1000 LEX centrifuge rotor (ThermoScientific) and resuspended in PBS, pH 7.2. DNase I was added and cell were lysed by three to four passages through a Microfluidizer M-110P (Microfluidics) at 25 kPa on ice. Cell debris was removed by centrifugation for 15 minutes at 4 °C and 10,000 g in a Sorvall SLA-1500 rotor (ThermoScientific). Membrane vesicles were harvested by ultracentrifugation for 2 h at 170,000 g in a Beckman Coulter ultracentrifuge using a Beckman Ti45 rotor and resuspended in 10 or 30 ml PBS, pH 7.2 supplemented with 10% glycerol. Prior to solubilization, 10 mM N-ethyl-maleimide was added and the resuspended membranes were rotated at 22 °C for 2 h. Then proteins were extracted by solubilizing the membranes using 1 % (w/v) β-DDM (Glycon Biochemicals) for 2 h at 4 °C. Unextracted material was harvested by ultracentrifugation at 170,000 g for 20 min and 0.5 ml of NHS activated Sepharose 4 Fast Flow (Cytiva) coupled to the anti-GFP nanobody (PDB-ID: 3K1K) was added to the supernatant to capture the GFP tagged MmpL4-MmpS4 complex. All purification steps were carried out at 4 °C. Batch binding was performed for 2 h, after which the resin was washed with 20 ml of PBS, 10 mM NEM, 10 % glycerol, 0.03 % β-DDM followed by 50 ml TBS (20 mM Tris/HCl, pH 7.5, 150 mM NaCl), 10% glycerol, 0.03% β-DDM. The resin was incubated with 100 μg 3C protease for 2 h to elute the protein by cleaving off the GFP-His_10_-tag. The flow through was collected and the resin was washed with 2-4 ml buffer to collect the cleaved protein. The sample was analyzed by reducing and non-reducing SDS-PAGE gel electrophoresis to assess crosslinking efficiency. Proteins were concentrated with 100 kDa MWCO concentrator (Merck Millipore) to 1-2 mg/ml and flash frozen and stored at −80 °C or immediately purified by size-exclusion chromatography on a Superose 6 Increase 10/300 GL column (Cytiva) in TBS, 0.03 % β-DDM.

### Cryo-EM sample preparation and data acquisition

The cross-linking construct #5 of the MmpS4-MmpL4 complex was concentrated to 4 mg/ml and applied to glow discharged (0.39 mbar, 15 mA, 1 min) holey carbon grids (QuantiFoil R1.2/1.3 Au-300 mesh). Excess sample was removed by blotting at 4 °C for 4.5-5.5 s with a relative humidity of 100% and a blotting force of 20 using a Vitrobot Mark IV (ThermoFisher). The sample was vitrified by plunging into ethane/propane. Cross-linked MmpS4-MmpL4 was imaged on a Titan Krios G3i (300 kV, 100 μm objective aperture) equipped with a BioQuantum energy filter (20 eV energy filter slit) and a K3 direct electron detector (6k x 4k pixels). EPU 2.9 was used for automated data acquisition of dose-fractionated micrographs in a defocus range of −1 – −2.2 μm in 0.2 μm increments. Micrographs were recorded at a magnification of 130,000x, with a pixel size of 0.65 Å and 1.3 s exposure time in counted super-resolution mode and Fourier cropped by 2. A total of 14588 micrographs were recorded for the cross-linked MmpL4-S4 complex #5 with a dose of 21.10 e^−^/px/sec resulting in a total electron exposure of 62.92 e^−^/Å^2^.

### Cryo-EM image processing, model building and refinement

The acquired data was processed in CryoSPARC4.7.0 [43]. Data quality was monitored and initial processing steps including patch motion correction and patch CTF estimation were performed in CryoSPARC live. Good micrographs were selected based on the resolution of the estimated CTF fit and relative ice thickness. Particles were selected and filtered in two different ways (Fig. S4). First, particles were picked using the blob picker, extracted with a box size of 400 pixel and Fourier cropped to 100 pixels (2.6 Å per pixel). Two rounds of 2D-classification were performed, followed by one ab-initio reconstruction and one additional round of 2D-classification. In a parallel approach, micrographs were first denoised and then particles were picked using the blob picker. The resulting particles were selected in the same way as described above, with two rounds of 2D-classification, one round of ab-initio reconstruction and one additional round of 2D-classification. The final selection of particles from both approaches were merged and duplicates were removed. Particles were unbinned, reextracted, and an ab-initio reconstruction was performed. The subsequent non-uniform (NU) refinement [44], was performed once with C1 symmetry and once with C3 symmetry constraints. NU refinement using C1 symmetry resulted in a 3.09 Å map of the MmpL4-S4 complex with a “short” CCD. NU refinement using C3 symmetry resulted in a 2.89 Å map of the MmpL4-S4 complex. The C1-symmetrical map was locally refined with a mask created from its CCD. The local refinement resulted in a 3.22 Å map of the MmpL4-S4 complex with a “long” CCD. Resolutions are stated according to the 0.143 cut-off criterion. Local resolution estimation was carried out in CryoSPARC.

### Model building

The monomeric MmpL4 model (PDB: 9GI0) was docked into the C3 symmetrical density obtained for the MmpL4-S4 complex using PHENIX [45], Coot [46] and ISOLDE [47]. This model was used as input for the C1-symmetrical model with “short” and “long” CCD and the AlphaFold3 prediction of the CCD of the MmpL4-S4 complex was docked into the tilted CCD density. Cavities of MmpL3, the monomeric MmpL4, and the MmpL4-S4 complex were calculated using a local installation of the 3 V package (shell probe radius: 5 Å, solvent-excluded probe radius of 1.4 A, grid spacing 1.3) [27].

### Mycobactin toxicity and bedaquiline susceptibility assays

The MmpS4-MmpL4-cys-depleted construct was analyzed in the mycobactin toxicity assay as described previously [9]. The susceptibility of the *Mtb*Δ*mmpL4/mmpL5* strain or the *MtbΔmmpL4/mmpL5* complemented with *mmpS4-mmpL4-cys-depleted* were measured using a microplate Alamar Blue assay (MABA). Briefly, cultures of the *Mtb*Δ*mmpL4/mmpL5* strain complemented with *mmpS4-mmpL4-cys-depleted* containing a C-terminal FLAG-tag were grown in supplemented 7H9 medium to an OD_600_ of 1. The cultures were then filtered using a 5 μM filter to remove clumps and cells were resuspended in Hartmans de Bont (HdB) minimal medium supplemented with 0.2% casamino acids, 24 μg/ml pantothenate, 20 μM hemin and 0.02% tyloxapol. The cells were then incubated in microplates with increasing mycobactin concentrations for five days at 37 °C. Resazurin (90 μM) was added and cells incubated for an additional six hours after which the fluorescence of the metabolically converted resazurin dye was measured as an indicator of cell viability.

## Supplementary Material

Supplement 1

## Data Availability

Structural models have been deposited to the Protein Data Bank (PDB) with the accession codes 9SYJ (data processed using C1 symmetry), 9SYT (data processed using C1 symmetry and applying a mask to improve the resolution of the CCD), and 9SYV (data processed using C3 symmetry). The cryo-EM data has been deposited in the Electron Microscopy Data Bank (EMDB) with the accession codes D_1292151303, D_1292151414, and D_1292151419 respectively. Plasmids and other data that support the findings of this study are available from the corresponding authors upon request.

## References

[1] Cole S.T., Brosch R., Parkhill J., Garnier T., Churcher C., Harris D., Gordon S.V., Eiglmeier K., Gas S., Barry C.E. 3rd, Tekaia F., Badcock K., Basham D., Brown D., Chillingworth T., Connor R., Davies R., Devlin K., Feltwell T., Gentles S., Hamlin N., Holroyd S., Hornsby T., Jagels K., Krogh A., McLean J., Moule S., Murphy L., Oliver K., Osborne J., Quail M.A., Rajandream M.A., Rogers J., Rutter S., Seeger K., Skelton J., Squares R., Squares S., Sulston J.E., Taylor K., Whitehead S., and Barrell B.G., Deciphering the biology of Mycobacterium tuberculosis from the complete genome sequence. Nature, 1998. 393(6685): p. 537–44. 9634230 10.1038/31159

[2] Xu Z.J., Meshcheryakov V.A., Poce G., and Chng S.S., MmpL3 is the flippase for mycolic acids in mycobacteria. Proc Natl Acad Sci U S A, 2017. 114(30): p. 7993–7998. 28698380 10.1073/pnas.1700062114 PMC5544280

[3] Domenech P., Reed M.B., and Barry C.E. 3rd, Contribution of the Mycobacterium tuberculosis MmpL protein family to virulence and drug resistance. Infect Immun, 2005. 73(6): p. 3492–501. 15908378 10.1128/IAI.73.6.3492-3501.2005 PMC1111821

[4] Adams O., Deme J.C., Parker J.L., Fowler P.W., Lea S.M., Newstead S., and Consortium C., Cryo-EM structure and resistance landscape of M. tuberculosis MmpL3: An emergent therapeutic target. Structure, 2021. 29(10): p. 1182–+. 34242558 10.1016/j.str.2021.06.013 PMC8752444

[5] Su C.C., Klenotic P.A., Cui M., Lyu M., Morgan C.E., and Yu E.W., Structures of the mycobacterial membrane protein MmpL3 reveal its mechanism of lipid transport. PLoS Biol, 2021. 19(8): p. e3001370. 34383749 10.1371/journal.pbio.3001370 PMC8384468

[6] Zhang B., Li J., Yang X., Wu L., Zhang J., Yang Y., Zhao Y., Zhang L., Yang X., Yang X., Cheng X., Liu Z., Jiang B., Jiang H., Guddat L.W., Yang H., and Rao Z., Crystal Structures of Membrane Transporter MmpL3, an Anti-TB Drug Target. Cell, 2019. 176(3): p. 636–648 e13. 30682372 10.1016/j.cell.2019.01.003

[7] North E.J., Schwartz C.P., Zgurskaya H.I., and Jackson M., Recent advances in mycobacterial membrane protein large 3 inhibitor drug design for mycobacterial infections. Expert Opinion on Drug Discovery, 2023. 18(7): p. 707–724. 37226498 10.1080/17460441.2023.2218082 PMC10330604

[8] Wells R.M., Jones C.M., Xi Z.Y., Speer A., Danilchanka O., Doornbos K.S., Sun P.B., Wu F.M., Tian C.L., and Niederweis M., Discovery of a Siderophore Export System Essential for Virulence of Mycobacterium tuberculosis. Plos Pathogens, 2013. 9(1). 10.1371/journal.ppat.1003120 PMC3561183 23431276

[9] Jones C.M., Wells R.M., Madduri A.V.R., Renfrow M.B., Ratledge C., Moody D.B., and Niederweis M., Self-poisoning of Mycobacterium tuberculosis by interrupting siderophore recycling. Proc Natl Acad Sci U S A, 2014. 111(5): p. 1945–1950. 24497493 10.1073/pnas.1311402111 PMC3918798

[10] Hartkoorn R.C., Uplekar S., and Cole S.T., Cross-Resistance between Clofazimine and Bedaquiline through Upregulation of MmpL5 in. Antimicrobial Agents and Chemotherapy, 2014. 58(5): p. 2979–2981. 24590481 10.1128/AAC.00037-14 PMC3993252

[11] Remm S., Earp J.C., Dick T., Dartois V., and Seeger M.A., Critical discussion on drug efflux in Mycobacterium tuberculosis. FEMS Microbiol Rev, 2022. 46(1). 10.1093/femsre/fuab050 PMC8829022 34637511

[12] Meikle V., Zhang L., and Niederweis M., Intricate link between siderophore secretion and drug efflux in. Antimicrobial Agents and Chemotherapy, 2023. 67(10). 10.1128/aac.01629-22 PMC10583673 37676015

[13] Van Rie A., Walker T., de Jong B., Rupasinghe P., Rivière E., Dartois V., Sonnenkalb L., Machado D., Gagneux S., Supply P., Dreyer V., Niemann S., Goig G., Meehan C., Tagliani E., and Cirillo D.M., Balancing access to BPaLM regimens and risk of resistance. Lancet Infectious Diseases, 2022. 22(10): p. 1411–1412. 36007529 10.1016/S1473-3099(22)00543-6

[14] Bernut A., Viljoen A., Dupont C., Sapriel G., Blaise M., Bouchier C., Brosch R., de Chastellier C., Herrmann J.L., and Kremer L., Insights into the smooth-to-rough transitioning in Mycobacterium bolletii unravels a functional Tyr residue conserved in all mycobacterial MmpL family members. Molecular Microbiology, 2016. 99(5): p. 866–883. 26585558 10.1111/mmi.13283

[15] Earp J.C., Garaeva A.A., Meikle V., Niederweis M., and Seeger M.A., Structural basis of siderophore export and drug efflux by Mycobacterium tuberculosis. Nat Commun, 2025. 16(1): p. 1934. 39994240 10.1038/s41467-025-56888-6 PMC11850643

[16] Xiong Z., Yang X., Wang S., Smart C.J., Sisson H.M., Lin Z., Hu T., Ran Y., Xu C., Yang X., Zhao Y., Jowsey W.J., Cook G.M., McNeil M.B., Guddat L.W., Rao Z., and Zhang B., Structure and assembly of the MmpL5/MmpS5 efflux transporter from Mycobacterium tuberculosis. Nat Commun, 2025. 16(1): p. 4976. 40442140 10.1038/s41467-025-60365-5 PMC12122801

[17] Seeger M.A., Schiefner A., Eicher T., Verrey F., Diederichs K., and Pos K.M., Structural asymmetry of AcrB trimer suggests a peristaltic pump mechanism. Science, 2006. 313(5791): p. 1295–8. 16946072 10.1126/science.1131542

[18] Maharjan R., Zhang Z.M., Klenotic P.A., Gregor W.D., Tringides M.L., Cui M., Purdy G.E., and Yu E.W., Structures of the mycobacterial MmpL4 and MmpL5 transporters provide insights into their role in siderophore export and iron acquisition. Plos Biology, 2024. 22(10). 10.1371/journal.pbio.3002874 PMC11524445 39423221

[19] Couston J., Feuillard J., Ancelin A., Lai-Kee-Him J., Brodolin K., Chalut C., Gourdon P., and Blaise M., A coiled-coil domain triggers oligomerization of MmpL10, the mycobacterial transporter of trehalose polyphleate precursor. FEBS Lett, 2025. 599(12): p. 1682–1697. 40468558 10.1002/1873-3468.70085 PMC12183618

[20] Yamamoto K., Nakata N., Mukai T., Kawagishi I., and Ato M., Coexpression of MmpS5 and MmpL5 Contributes to Both Efflux Transporter MmpL5 Trimerization and Drug Resistance in. Msphere, 2021. 6(1). 10.1128/mSphere.00518-20 PMC7845600 33408221

[21] Zhang Z.M., Maharjan R., Gregor W.D., Klenotic P.A., and Yu E.W., Structures of MmpL complexes reveal the assembly and mechanism of this family of transporters. Science Advances, 2025. 11(33). 10.1126/sciadv.adx1129 PMC12346298 40802754

[22] Fountain A.J., Bohning J., McLaughlin S.H., Morgan T.E., Edelstein P.H., Troll M., Lamers M.H., Bharat T.A.M., Luisi B.F., and Ramakrishnan L., Structural and functional analysis of the Mycobacterium tuberculosis MmpS5L5 efflux pump presages increased bedaquiline resistance. Proc Natl Acad Sci U S A, 2025. 122(39): p. e2516660122. 40986343 10.1073/pnas.2516660122 PMC12501195

[23] Maharjan R., Zhang Z., Klenotic P.A., Gregor W.D., Purdy G.E., and Yu E.W., Cryo-EM structure of the Mycobacterium smegmatis MmpL5-AcpM complex. mBio, 2024. 15(12): p. e0303524. 39480109 10.1128/mbio.03035-24 PMC11633376

[24] Jumper J., Evans R., Pritzel A., Green T., Figurnov M., Ronneberger O., Tunyasuvunakool K., Bates R., Zidek A., Potapenko A., Bridgland A., Meyer C., Kohl S.A.A., Ballard A.J., Cowie A., Romera-Paredes B., Nikolov S., Jain R., Adler J., Back T., Petersen S., Reiman D., Clancy E., Zielinski M., Steinegger M., Pacholska M., Berghammer T., Bodenstein S., Silver D., Vinyals O., Senior A.W., Kavukcuoglu K., Kohli P., and Hassabis D., Highly accurate protein structure prediction with AlphaFold. Nature, 2021. 596(7873): p. 583–589. 34265844 10.1038/s41586-021-03819-2 PMC8371605

[25] Cuthbert B.J., Mendoza J., de Miranda R., Papavinasasundaram K., Sassetti C.M., and Goulding C.W., The structure of Mycobacterium thermoresistibile MmpS5 reveals a conserved disulfide bond across mycobacteria. Metallomics, 2024. 16(3). 10.1093/mtomcs/mfae011 PMC10929441 38425033

[26] Seeger M.A., von Ballmoos C., Eicher T., Brandstatter L., Verrey F., Diederichs K., and Pos K.M., Engineered disulfide bonds support the functional rotation mechanism of multidrug efflux pump AcrB. Nat Struct Mol Biol, 2008. 15(2): p. 199–205. 18223659 10.1038/nsmb.1379

[27] Voss N.R. and Gerstein M., 3V: cavity, channel and cleft volume calculator and extractor. Nucleic Acids Res, 2010. 38(Web Server issue): p. W555–62. 20478824 10.1093/nar/gkq395 PMC2896178

[28] Seeger M.A., von Ballmoos C., Verrey F., and Pos K.M., Crucial role of Asp408 in the proton translocation pathway of multidrug transporter AcrB: evidence from site-directed mutagenesis and carbodiimide labeling. Biochemistry, 2009. 48(25): p. 5801–12. 19425588 10.1021/bi900446j

[29] Eicher T., Seeger M.A., Anselmi C., Zhou W., Brandstatter L., Verrey F., Diederichs K., Faraldo-Gomez J.D., and Pos K.M., Coupling of remote alternating-access transport mechanisms for protons and substrates in the multidrug efflux pump AcrB. Elife, 2014. 3. 10.7554/eLife.03145 PMC4359379 25248080

[30] Pravda L., Sehnal D., Tousek D., Navratilova V., Bazgier V., Berka K., Svobodova Varekova R., Koca J., and Otyepka M., MOLEonline: a web-based tool for analyzing channels, tunnels and pores (2018 update). Nucleic Acids Res, 2018. 46(W1): p. W368–W373. 29718451 10.1093/nar/gky309 PMC6030847

[31] Chen J., Fruhauf A., Fan C., Ponce J., Ueberheide B., Bhabha G., and Ekiert D.C., Structure of an endogenous mycobacterial MCE lipid transporter. Nature, 2023. 620(7973): p. 445–452. 37495693 10.1038/s41586-023-06366-0 PMC12817965

[32] Mosalaganti S., Obarska-Kosinska A., Siggel M., Taniguchi R., Turonova B., Zimmerli C.E., Buczak K., Schmidt F.H., Margiotta E., Mackmull M.T., Hagen W.J.H., Hummer G., Kosinski J., and Beck M., AI-based structure prediction empowers integrative structural analysis of human nuclear pores. Science, 2022. 376(6598): p. eabm9506. 35679397 10.1126/science.abm9506

[33] Andersen C., Koronakis E., Bokma E., Eswaran J., Humphreys D., Hughes C., and Koronakis V., Transition to the open state of the TolC periplasmic tunnel entrance. Proc Natl Acad Sci U S A, 2002. 99(17): p. 11103–8. 12163644 10.1073/pnas.162039399 PMC123217

[34] Zhang L., Kent J.E., Whitaker M., Young D.C., Herrmann D., Aleshin A.E., Ko Y.H., Cingolani G., Saad J.S., Moody D.B., Marassi F.M., Ehrt S., and Niederweis M., A periplasmic cinched protein is required for siderophore secretion and virulence of. Nature Communications, 2022. 13(1). 10.1038/s41467-022-29873-6 PMC9042941 35474308

[35] Eicher T., Cha H.J., Seeger M.A., Brandstatter L., El-Delik J., Bohnert J.A., Kern W.V., Verrey F., Grütter M.G., Diederichs K., and Pos K.M., Transport of drugs by the multidrug transporter AcrB involves an access and a deep binding pocket that are separated by a switch-loop. Proc Natl Acad Sci U S A, 2012. 109(15): p. 5687–92. 22451937 10.1073/pnas.1114944109 PMC3326505

[36] Zwama M., Yamasaki S., Nakashima R., Sakurai K., Nishino K., and Yamaguchi A., Multiple entry pathways within the efflux transporter AcrB contribute to multidrug recognition. Nature Communications, 2018. 9. 10.1038/s41467-017-02493-1 PMC5760665 29317622

[37] Tam H.K., Foong W.E., Oswald C., Herrmann A., Zeng H., and Pos K.M., Allosteric drug transport mechanism of multidrug transporter AcrB. Nat Commun, 2021. 12(1): p. 3889. 34188038 10.1038/s41467-021-24151-3 PMC8242077

[38] Murakami S., Nakashima R., Yamashita E., Matsumoto T., and Yamaguchi A., Crystal structures of a multidrug transporter reveal a functionally rotating mechanism. Nature, 2006. 443(7108): p. 173–9. 16915237 10.1038/nature05076

[39] Zhong B., Su X., Wen M., Zuo S., Hong L., and Lin J., ParaFold: Paralleling AlphaFold for Large-Scale Predictions, in International Conference on High Performance Computing in Asia-Pacific Region Workshops. 2022, Association for Computing Machinery: Virtual Event, Japan. p. 1–9.

[40] Abramson J., Adler J., Dunger J., Evans R., Green T., Pritzel A., Ronneberger O., Willmore L., Ballard A.J., Bambrick J., Bodenstein S.W., Evans D.A., Hung C.C., O'Neill M., Reiman D., Tunyasuvunakool K., Wu Z., Zemgulyte A., Arvaniti E., Beattie C., Bertolli O., Bridgland A., Cherepanov A., Congreve M., Cowen-Rivers A.I., Cowie A., Figurnov M., Fuchs F.B., Gladman H., Jain R., Khan Y.A., Low C.M.R., Perlin K., Potapenko A., Savy P., Singh S., Stecula A., Thillaisundaram A., Tong C., Yakneen S., Zhong E.D., Zielinski M., Zidek A., Bapst V., Kohli P., Jaderberg M., Hassabis D., and Jumper J.M., Accurate structure prediction of biomolecular interactions with AlphaFold 3. Nature, 2024. 630(8016): p. 493–500. 38718835 10.1038/s41586-024-07487-w PMC11168924

[41] Geertsma E.R., FX cloning: a simple and robust high-throughput cloning method for protein expression. Methods Mol Biol, 2014. 1116: p. 153–64. 24395363 10.1007/978-1-62703-764-8_11

[42] Geertsma E.R. and Dutzler R., A versatile and efficient high-throughput cloning tool for structural biology. Biochemistry, 2011. 50(15): p. 3272–8. 21410291 10.1021/bi200178z

[43] Punjani A., Rubinstein J.L., Fleet D.J., and Brubaker M.A., cryoSPARC: algorithms for rapid unsupervised cryo-EM structure determination. Nat Methods, 2017. 14(3): p. 290–296. 28165473 10.1038/nmeth.4169

[44] Punjani A., Zhang H., and Fleet D.J., Non-uniform refinement: adaptive regularization improves single-particle cryo-EM reconstruction. Nat Methods, 2020. 17(12): p. 1214–1221. 33257830 10.1038/s41592-020-00990-8

[45] Liebschner D., Afonine P.V., Baker M.L., Bunkoczi G., Chen V.B., Croll T.I., Hintze B., Hung L.W., Jain S., McCoy A.J., Moriarty N.W., Oeffner R.D., Poon B.K., Prisant M.G., Read R.J., Richardson J.S., Richardson D.C., Sammito M.D., Sobolev O.V., Stockwell D.H., Terwilliger T.C., Urzhumtsev A.G., Videau L.L., Williams C.J., and Adams P.D., Macromolecular structure determination using X-rays, neutrons and electrons: recent developments in Phenix. Acta Crystallogr D Struct Biol, 2019. 75(Pt 10): p. 861–877. 31588918 10.1107/S2059798319011471 PMC6778852

[46] Emsley P. and Cowtan K., Coot: model-building tools for molecular graphics. Acta Crystallogr D Biol Crystallogr, 2004. 60(Pt 12 Pt 1): p. 2126–32. 15572765 10.1107/S0907444904019158

[47] Croll T.I., ISOLDE: a physically realistic environment for model building into low-resolution electron-density maps. Acta Crystallogr D Struct Biol, 2018. 74(Pt 6): p. 519–530. 29872003 10.1107/S2059798318002425 PMC6096486

