# Supplementary material for: Structural elucidation of the hexameric MmpS4-MmpL4 complex from Mycobacterium tuberculosis
Source: bioRxiv. 2026 Jan 7:2026.01.07.698164. Preprint. [Version 1] doi: 10.64898/2026.01.07.698164 (PMC12803245; doi:10.64898/2026.01.07.698164)
Supplement: Supplement 1 [file NIHPP2026.01.07.698164v1-supplement-1.pdf]

bioRxiv preprint doi: https://doi.org/10.64898/2026.01.07.698164; this version posted January 8, 2026. The copyright holder for this preprint (which was not certified by peer review) is the author/funder, who has granted bioRxiv a license to display the preprint in perpetuity. It is made available under aCC-BY 4.0 International license.

SUPPLEMENTARY INFORMATION

Table S1: Cryo-electron microscopy data collection statistics

	MmpS4-MmpL4 "C3 symmetrical" (EMD-55355, PDB: 9SYV)	MmpS4-MmpL4 "short CCD" (EMD-55350, PDB: 9SYJ)	MmpS4-MmpL4 "long CCD" (EMD-55353, PDB: 9SYT)
**Data Collection & Processing**			
Microscope	Titan Krios G3i		
Camera	Gatan K3 GIF		
Magnification	130,000		
Voltage (kV)	300		
Electron exposure ($e^-/Å^2$)	62.92		
Defocus range (µm)	-1 to -2.2		
Pixel Size (Å)	0.65		
Initial number of micrographs (no.)	14,588		
Initial Particle Images (no.)	8,987,065		
Final Particle Images (no.)	76,903	76,903	76,903
Symmetry Imposed	C3	C1	C1
Map Resolution (Å)	2.89	3.09	3.22
FSC Threshold	0.143	0.143	0.143
Map Resolution Range (Å)	2.8 – 4.0	2.9 – 6.5	3.0 – 8.0
**Refinement**			
Initial Model Used	9GI0 and AF model	9GI0 and AF model	9GI0 and AF model
Model Resolution (Å)	2.9	3.1	3.2
FSC Threshold	0.143	0.143	0.143
Model Resolution Range (Å)	2.8 – 4.0	2.9 – 6.5	3.0 – 8.0
Map sharpening b-factor ($Å^2$)	-78.2	-62.6	-63.9
**Model Composition**			
Non-hydrogen Atoms	35,235	37,508	38,803
Protein Residues	2,274	2,422	2,507
Ligands	LMT: 3	LMT: 3	LMT: 3
B Factor ($Å^2$)			

bioRxiv preprint doi: https://doi.org/10.64898/2026.01.07.698164; this version posted January 8, 2026. The copyright holder for this preprint (which was not certified by peer review) is the author/funder, who has granted bioRxiv a license to display the preprint in perpetuity. It is made available under aCC-BY 4.0 International license.

Protein	50.34	66.47	153.81
Ligand	56.69	67.39	124.06
R.m.s deviations			
Bond Lengths (Å)	0.004	0.004	0.004
Bond Angles (°)	0.716	0.630	0.653
**Validation**			
MolProbity Score	0.94	1.01	1.16
Clashscore	1.79	2.35	2.73
Poor Rotamers (%)	0.50	0.00	0.00
**Ramachandran Plot**			
Favoured (%)	98.01	98.33	97.51
Allowed (%)	1.99	1.67	2.49
Disallowed (%)	0	0	0

Table S2: Nucleotide sequence of *mmpS4-mmpL4_cys-depleted*

**Sequence name**	**Sequence (5'-3')**
mmpS4-mmpL4-cysteine-depleted	CTAATGCGGACTTGGATTCCACTGGTCATCCTGGTGGTGGTCATCGTCGGGGGCTTCACCGTGCACCGGATCCGCGG<br>CTTCTTCGGCTCCGAAAACCGCCCGTCGTACTCCGACACCAACCTGGAAAACAGCAAACCATTCAACCCTAAACACCT<br>GACTTACGAGATCTTCGGACCCCCCGGAACAGTCGCCGACATTAGTTATTTCGACGTCAATTCCGAGCCGCAACGGG<br>TCGATGGAGCGGTGCTACCGTGGTCATTGCATATCACGACAAACGACGCGGCGGTGATGGGAAATATCGTGGCACA<br>AGGTAATAGCGACAGCATTGGCTGCCGAATCACGGTAGACGGCAAGGTCAGGGCCGAGAGGGTTTCCAACGAAGT<br>CAACGCCTATACTTACTGCTTGGTGAAGTCCGCGTGAGTACTAAATTCGCGAACGACTCCAATACCAACGCTCGCCCC<br>GAAAAGCCATTCATCGCCAGGATGATCCACGCCTTTGCGGTACCGATCATCCTTGGCTGGCTGGCAGTTTCCGTCGT<br>TGTCACCGTATTTGTCCCGTCACTGGAAGCTGTCGGCCAAGAGCGATCGGTGTCGCTGAGTCCCAAGGATGCACCGT<br>CGTTTGAGGCGATGGGACGTATCGGCATGGTGTTCAAGGAAGGCGATTCCGACAGTTTCGCGATGGTCATAATAGA<br>GGGTAACCAACCCCTTGGCGACGCTGCCCATAAGTATTACGACGGCCTGGTTGCTCAATTGAGGGCCGATAAGAAG<br>CACGTGCAAAGTGTCCAAGATTTATGGGGGGACCCACTCACCGCCGCGGGCGTGCAAAGTAACGACGGCAAGGCC<br>GCCTATGTTCAACTGTCACTTGCCGGCAACCAAGGCACGCCGCTGGCCAACGAATCCGTCGAGGCAGTACGCAGCA<br>TCGTCGAAAGCACGCCCGCGCCGCCGGGCATAAAGGCCTATGTGACCGGACCATCCGCACTCGCCGCGGATATGCA<br>CCACAGTGGCGATAGATCCATGGCCAGGATCACCATGGTTACGGTCGCCGTGATCTTTATTATGTTGTTGCTCGTCTA<br>CCGGTCGATAATCACCGTGGTTCTCCTGCTGATCACGGTGGGGGTCGAATTGACGGCTGCGCGCGGAGTCGTAGCG<br>GTTCTGGGGCATAGCGGGGCTATCGGACTTACCACCTTTGCGGTGAGCTTGCTGACTTCGCTGGCAATCGCGGCCG<br>GTACGGACTACGGGATATTCATCATCGGGCGCTACCAGGAAGCCCGCCAAGCCGGCGAGGACAAAGAGGCCGCCT<br>ACTACACCATGTACCGCGGGACCGCCCACGTGATTCTGGGCTCTGGATTGACCATCGCCGGAGCTACCTTTTCCCTG<br>AGCTTTGCCCGCATGCCCTACTTTCAAACCCTGGGCATCCCCTCCGCGGTGGGGATGCTAGTCGCAGTGGCGGTCGC<br>GTTGACGCTGGGTCCGGCCGTCCTACACGTCGGCAGCCGGTTCGGCCTGTTCGACCCTAAGCGGCTCCTCAAAGTCC<br>GTGGCTGGCGGCGGGTGGGTACCGTGGTGGTTCGCTGGCCGCTGCCCGTTCTCGTCGCCACCTCCGCGATCGCCCT<br>AGTCGGTCTGCTCGCCCTGCCTGGATACAAAACCAGCTACAACGACCGTGACTACCTACCGGACTTCATACCCGCCA<br>ACCAAGGATATGCGGCCGCGGATCGTCATTTCTCTCAGGCCAGGATGAAGCCCGAGATCCTAATGATTGAATCCGAT<br>CACGATATGCGGAATCCGGCAGATTTTCTGGTCCTGGACAAGCTGGCTAAAGGCATCTTCCGGGTCCCGGGCATTTC<br>CCGTGTGCAAGCAATTACCCGGCCCGAAGGAACGACGATGGACCACACGTCGATCCCGTTCCAGATCAGCATGCAA<br>AATGCCGGTCAGCTGCAGACCATAAAGTACCAGCGCGACCGGGCGAACGACATGCTGAAGCAGGCCGACGAGATG<br>GCCACGACGATTGCGGTGTTGACGCGGATGCATAGCTTGATGGCGGAGATGGCCAGTACTACCCACCGCATGGTCG

bioRxiv preprint doi: https://doi.org/10.64898/2026.01.07.698164; this version posted January 8, 2026. The copyright holder for this preprint (which was not certified by peer review) is the author/funder, who has granted bioRxiv a license to display the preprint in perpetuity. It is made available under aCC-BY 4.0 International license.

GCGACACCGAAGAGATGAAGGAGATCACCGAAGAATTACGCGACCACGTCGCGGATTTTGATGATTTCTGGAGACC
AATTCGAAGCTATTTCTACTGGGAAAAGCACTGCTACGGAATTCCGATCTGTTGGTCGTTCAGATCGATATTCGATGC
ACTGGACGGAATCGACAAGCTCAGCGAGCAAATAGGCGTCCTCTTGGGCGACTTGCGCGAGATGGATCGCCTCATG
CCACAAATGGTCGCGCAGATCCCGCCGCAGATTGAGGCCATGGAAAACATGCGGACCATGATTCTTACCATGCACA
GCACCATGACCGGAATCTTCGACCAGATGCTCGAAATGAGCGACAATGCCACAGCCATGGGTAAGGCTTTTGACGC
CGCCAAGAACGACGACTCGTTCTACCTTCCACCCGAGGTTTTCAAGAACAAAGACTTCCAGCGCGCCATGAAGTCGT
TCCTGTCTTCGGACGGACACGCGGCCCGGTTTATCATCCTGCACAGGGGGGATCCGCAATCACCCGAGGGCATCAA
AAGTATCGACGCGATTCGTACCGCGGCCGAGGAATCGCTCAAGGGAACTCCACTAGAAGACGCCAAGATCTACCTG
GCCGGCACGGCGGCCGTCTTCCACGACATCTCCGAGGGCGCCCAATGGGACCTTCTTATCGCGGCAATTTCGTCCCT
CTCTCTCATTTTTATCATCATGCTGATCATCACACGGGCCTTTATTGCCGCCGCCGTCATTGTGGGCACAGTCGCACTT
TCACTGGGTGCCTCTTTCGGACTATCGGTGCTGCTCTGGCAGCATATTCTCGCAATCCATCTGCATTGGCTCGTGCTT
GCGATGTCGGTCATCGTTCTGTTGGCGGTGGGATCTGACTACAATCTGCTCTTGGTCTCCCGGTTCAAACAGGAAAT
AGGAGCGGGGCTGAAGACCGGCATCATCCGGTCGATGGGCGGCACCGGCAAGGTCGTGACCAACGCAGGGCTGG
TATTCGCCGTCACCATGGCGTCCATGGCCGTCAGCGATCTGCGGGTTATCGGACAGGTGGGCACCACCATCGGTCTG
GGTTTGCTGTTCGACACGTTGATCGTGCGATCGTTCATGACGCCATCCATCGCCGCACTGCTGGGCCGTTGGTTTTG
GTGGCCGCTGCGCGTTCGCAGCCGTCCAGCCCGTACCCCGACCGTGCCGAGTGAAACGCAACCGGCGGGTCGTCCG
TTAGCGATGAGCAGCGATCGTCTGGGTGAGTCTAATGCGGACTTGGATTCCACTGGTCATCCTGGTGGTGGTCATCG
TCGGGGGCTTCACCGTGCACCGGATCCGCGGCTTCTTCGGCTCCGAAAACCGCCCGTCGTACTCCGACACCAACCTG
GAAAACAGCAAACCATTCAACCCTAAACACCTGACTTACGAGATCTTCGGACCCCCCGGAACAGTCGCCGACATTAG
TTATTTCGACGTCAATTCCGAGCCGCAACGGGTCGATGGAGCGGTGCTACCGTGGTCATTGCATATCACGACAAACG
ACGCGGCGGTGATGGGAAATATCGTGGCACAAGGTAATAGCGACAGCATTGGCTGCCGAATCACGGTAGACGGCA
AGGTCAGGGCCGAGAGGGTTTCCAACGAAGTCAACGCCTATACTTACTGCTTGGTGAAGTCCGCGTGAGTACTAAA
TTCGCGAACGACTCCAATACCAACGCTCGCCCCGAAAAGCCATTCATCGCCAGGATGATCCACGCCTTTGCGGTACC
GATCATCCTTGGCTGGCTGGCAGTTTCCGTCGTTGTCACCGTATTTGTCCCGTCACTGGAAGCTGTCGGCCAAGAGC
GATCGGTGTCGCTGAGTCCCAAGGATGCACCGTCGTTTGAGGCGATGGGACGTATCGGCATGGTGTTCAAGGAAG
GCGATTCCGACAGTTTCGCGATGGTCATAATAGAGGGTAACCAACCCCTTGGCGACGCTGCCCATAAGTATTACGAC
GGCCTGGTTGCTCAATTGAGGGCCGATAAGAAGCACGTGCAAAGTGTCCAAGATTTATGGGGGGACCCACTCACCG
CCGCGGGCGTGCAAAGTAACGACGGCAAGGCCGCCTATGTTCAACTGTCACTTGCCGGCAACCAAGGCACGCCGCT
GGCCAACGAATCCGTCGAGGCAGTACGCAGCATCGTCGAAAGCACGCCCGCGCCGCCGGGCATAAAGGCCTATGT
GACCGGACCATCCGCACTCGCCGCGGATATGCACCACAGTGGCGATAGATCCATGGCCAGGATCACCATGGTTACG
GTCGCCGTGATCTTTATTATGTTGTTGCTCGTCTACCGGTCGATAATCACCGTGGTTCTCCTGCTGATCACGGTGGGG
GTCGAATTGACGGCTGCGCGCGGAGTCGTAGCGGTTCTGGGGCATAGCGGGGCTATCGGACTTACCACCTTTGCGG
TGAGCTTGCTGACTTCGCTGGCAATCGCGGCCGGTACGGACTACGGGATATTCATCATCGGGCGCTACCAGGAAGC
CCGCCAAGCCGGCGAGGACAAAGAGGCCGCCTACTACACCATGTACCGCGGGACCGCCCACGTGATTCTGGGCTCT
GGATTGACCATCGCCGGAGCTACCTTTTCCCTGAGCTTTGCCCGCATGCCCTACTTTCAAACCCTGGGCATCCCCTCC
GCGGTGGGGATGCTAGTCGCAGTGGCGGTCGCGTTGACGCTGGGTCCGGCCGTCCTACACGTCGGCAGCCGGTTC
GGCCTGTTCGACCCTAAGCGGCTCCTCAAAGTCCGTGGCTGGCGGCGGGTGGGTACCGTGGTGGTTCGCTGGCCGC
TGCCCGTTCTCGTCGCCACCTCCGCGATCGCCCTAGTCGGTCTGCTCGCCCTGCCTGGATACAAAACCAGCTACAACG
ACCGTGACTACCTACCGGACTTCATACCCGCCAACCAAGGATATGCGGCCGCGGATCGTCATTTCTCTCAGGCCAGG
ATGAAGCCCGAGATCCTAATGATTGAATCCGATCACGATATGCGGAATCCGGCAGATTTTCTGGTCCTGGACAAGCT
GGCTAAAGGCATCTTCCGGGTCCCGGGCATTTCCCGTGTGCAAGCAATTACCCGGCCCGAAGGAACGACGATGGAC
CACACGTCGATCCCGTTCCAGATCAGCATGCAAAATGCCGGTCAGCTGCAGACCATAAAGTACCAGCGCGACCGGG
CGAACGACATGCTGAAGCAGGCCGACGAGATGGCCACGACGATTGCGGTGTTGACGCGGATGCATAGCTTGATGG
CGGAGATGGCCAGTACTACCCACCGCATGGTCGGCGACACCGAAGAGATGAAGGAGATCACCGAAGAATTACGCG
ACCACGTCGCGGATTTTGATGATTTCTGGAGACCAATTCGAAGCTATTTCTACTGGGAAAAGCACTGCTACGGAATT
CCGATCTGTTGGTCGTTCAGATCGATATTCGATGCACTGGACGGAATCGACAAGCTCAGCGAGCAAATAGGCGTCCT
CTTGGGCGACTTGCGCGAGATGGATCGCCTCATGCCACAAATGGTCGCGCAGATCCCGCCGCAGATTGAGGCCATG
GAAAACATGCGGACCATGATTCTTACCATGCACAGCACCATGACCGGAATCTTCGACCAGATGCTCGAAATGAGCG
ACAATGCCACAGCCATGGGTAAGGCTTTTGACGCCGCCAAGAACGACGACTCGTTCTACCTTCCACCCGAGGTTTTC
AAGAACAAAGACTTCCAGCGCGCCATGAAGTCGTTCCTGTCTTCGGACGGACACGCGGCCCGGTTTATCATCCTGCA
CAGGGGGGATCCGCAATCACCCGAGGGCATCAAAAGTATCGACGCGATTCGTACCGCGGCCGAGGAATCGCTCAA
GGGAACTCCACTAGAAGACGCCAAGATCTACCTGGCCGGCACGGCGGCCGTCTTCCACGACATCTCCGAGGGCGCC
CAATGGGACCTTCTTATCGCGGCAATTTCGTCCCTCTCTCTCATTTTTATCATCATGCTGATCATCACACGGGCCTTTAT
TGCCGCCGCCGTCATTGTGGGCACAGTCGCACTTTCACTGGGTGCCTCTTTCGGACTATCGGTGCTGCTCTGGCAGC
ATATTCTCGCAATCCATCTGCATTGGCTCGTGCTTGCGATGTCGGTCATCGTTCTGTTGGCGGTGGGATCTGACTACA
ATCTGCTCTTGGTCTCCCGGTTCAAACAGGAAATAGGAGCGGGGCTGAAGACCGGCATCATCCGGTCGATGGGCGG
CACCGGCAAGGTCGTGACCAACGCAGGGCTGGTATTCGCCGTCACCATGGCGTCCATGGCCGTCAGCGATCTGCGG
GTTATCGGACAGGTGGGCACCACCATCGGTCTGGGTTTGCTGTTCGACACGTTGATCGTGCGATCGTTCATGACGCC
ATCCATCGCCGCACTGCTGGGCCGTTGGTTTTGGTGGCCGCTGCGCGTTCGCAGCCGTCCAGCCCGTACCCCGACCG
TGCCGAGTGAAACGCAACCGGCGGGTCGTCCGTTAGCGATGAGCAGCGATCGTCTGGGT

bioRxiv preprint doi: https://doi.org/10.64898/2026.01.07.698164; this version posted January 8, 2026. The copyright holder for this preprint (which was not certified by peer review) is the author/funder, who has granted bioRxiv a license to display the preprint in perpetuity. It is made available under aCC-BY 4.0 International license.

521 Table S3: Primers used in this study.

**Primer name**	**Sequence (5'-3')**
mmpS4_ W6_Fw	AATGCGGACTTGTATTCCACTGGTCATCC
mmpS4_W6_Rv	CCAGTGGAATACAAGTCCGCATTAGACTC
mmpS4_F29_Fw	CGCGGCTTCTGCGGCTCCGAAAACC
mmpS4_F29_Rv	CGGAGCCGCAGAAGCCGCGGATCC
mmpS4_Y37_Fw	GCCCGTCGTGCTCCGACACCAACCTG
mmpS4_Y37_Rv	GTGTCGGAGCACGACGGGCGGTTTTCG
mmpS4_D39_Fw	GTCGTACTCCTGCACCAACCTGGAAAACAGC
mmpS4_D39_Rv	CAGGTTGGTGCAGGAGTACGACGGGC
mmpS4_N44_Fw	CAACCTGGAATGCAGCAAACCATTCAACCC
mmpS4_N44_Rv	ATGGTTTGCTGCATTCCAGGTTGGTGTCGG
mmpS4_A95_Fw	GACAAACGACTGCGCGGTGATGGGAAATATC
mmpS4_A95_Rv	CATCACCGCGCAGTCGTTTGTCGTGATATG
mmpS4_S139_Fw	GGTGAAGTGCGCGTGAGTACTAAATTCGC
mmpS4_S139_Rv	GTACTCACGCGCACTTCACCAAGCAGTAAG
mmpL4_P935_Fw	TTGGTGGTGTCTGCGCGTTCGCAGC
mmpL4_P935_Rv	CGCGCAGACACCACCAAAACCAACGGC
mmpL4_R324_Fw	GCTTTGCCTGCATGCCCTACTTTCAAACC
mmpL4_R324_Rv	GTAGGGCATGCAGGCAAAGCTCAGGGAAAAG
mmpL4_D64_Fw	GTCCCAAGTGTGCACCGTCGTTTGAGG
mmpL4_D64_Rv	GACGGTGCACACTTGGGACTCAGCG
mmpL4_K742_Fw	GAATCGCTCTGTGGAACTCCACTAGAAG
mmpL4_K742_Rv	GGAGTTCCACAGAGCGATTCCTCGG
mmpL4_S434_Fw	GTCATTTCTGTCAGGCCAGGATGAAGCCC
mmpL4_S434_Rv	CCTGGCCTGACAGAAATGACGATCCGC

bioRxiv preprint doi: https://doi.org/10.64898/2026.01.07.698164; this version posted January 8, 2026. The copyright holder for this preprint (which was not certified by peer review) is the author/funder, who has granted bioRxiv a license to display the preprint in perpetuity. It is made available under aCC-BY 4.0 International license.

mmpL4_E483_Fw	CCGGCCCTGTGGAACGACGATGGACC
mmpL4_E483_Rv	CGTCGTTCCACAGGGCCGGGTAATTGC
mmpL4_M487_Fw	GGAACGACGTGTGACCACACGTCGATCC
mmpL4_M487_Rv	CGTGTGGTCACACGTCGTTCCTTCGGG
mmpL4_V690_Fw	CCACCCGAGTGTTTCAAGAACAAAGACTTC
mmpL4_V690_Rv	GTTCTTGAAACACTCGGGTGGAAGGTAG
FLAG_Fw	AAA GAT CAT GAT ATC GAT TAC AAG GAT GAC GAT GAC AAG TGA GAA GCT TTA GCT AAT TAA TTG GGG
FLAG_Rv	ATATCATGATCTTTATAATCACCGTCATGGTCTTTGTAGTCgccgaggcggtcgctg
pML4324_backbone_Fw	GCA GCG ATC GTC TGG GTG ACT ACA AAG ACC ATG ACG G
pML4324_backbone_Rv	GAA TCC AAG TCC GCA TTA GCA CTA TTA ACC TCC TTT CTG TTA ATT AAG C
mmpS4L4_Fw	TAA CAG AAA GGA GGT TAA TAG TGC TAA TGC GGA CTT GGA TTC
mmpS4L4_Rv	CAT GGT CTT TGT AGT CAC CCA GAC GAT CGC TGC

bioRxiv preprint doi: https://doi.org/10.64898/2026.01.07.698164; this version posted January 8, 2026. The copyright holder for this preprint (which was not certified by peer review) is the author/funder, who has granted bioRxiv a license to display the preprint in perpetuity. It is made available under aCC-BY 4.0 International license.

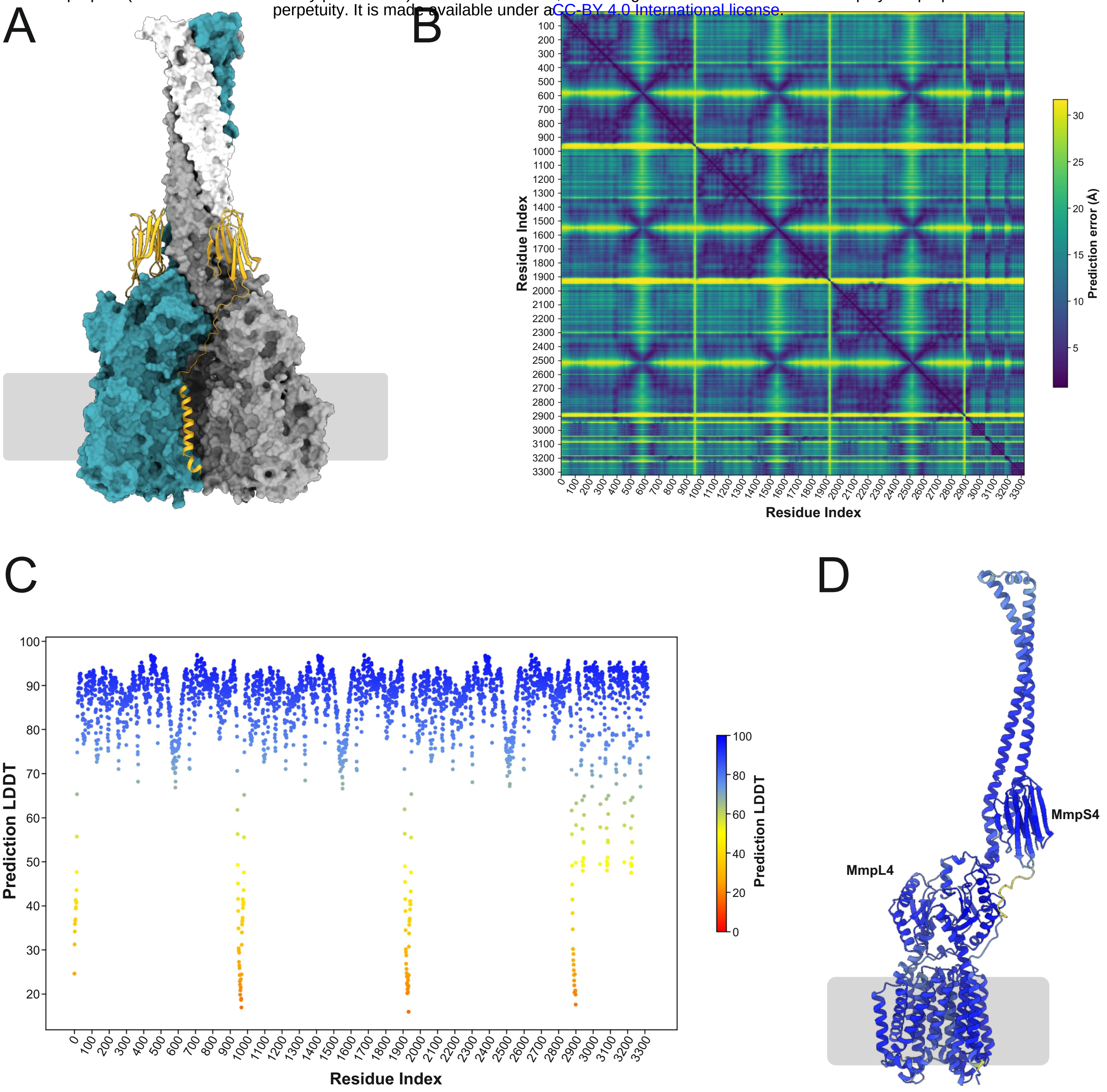

Supplementary Figure 1 | AlphaFold2 prediction of the hexameric (MmpS4)$_3$-(MmpL4)$_3$ complex. (**A**) The three protomers of MmpL4 are shown in surface presentation and colored petrol, grey and white. The MmpS4 protomers are shown as yellow cartoon (only two of them are visible). The boundary of the cytoplasmic membrane is indicated by grey lines. (**B**) Predicted alignment error (PAE) plot of the hexameric (MmpS4)$_3$-(MmpL4)$_3$ complex reported by AlphaFold2. It shows the expected distance error (in Å) between each residue in the predicted model. The three MmpL4 protomers cover residues 1-2901, and the three MmpS4 protomers residues 2902-3321. (**C**) Predicted Local Distance Difference Test (pLDDT) plot. The three MmpL4 protomers cover residues 1-2901, and the three MmpS4 protomers residues 2902-3321. (**D**) Depiction of pLDDT values in the context of the predicted MmpL4/MmpS4 structure (only first protomer shown, N- and C-terminus of MmpL4 cleaved).

bioRxiv preprint doi: https://doi.org/10.64898/2026.01.07.698164; this version posted January 8, 2026. The copyright holder for this preprint (which was not certified by peer review) is the author/funder, who has granted bioRxiv a license to display the preprint in perpetuity. It is made available under aCC-BY 4.0 International license.

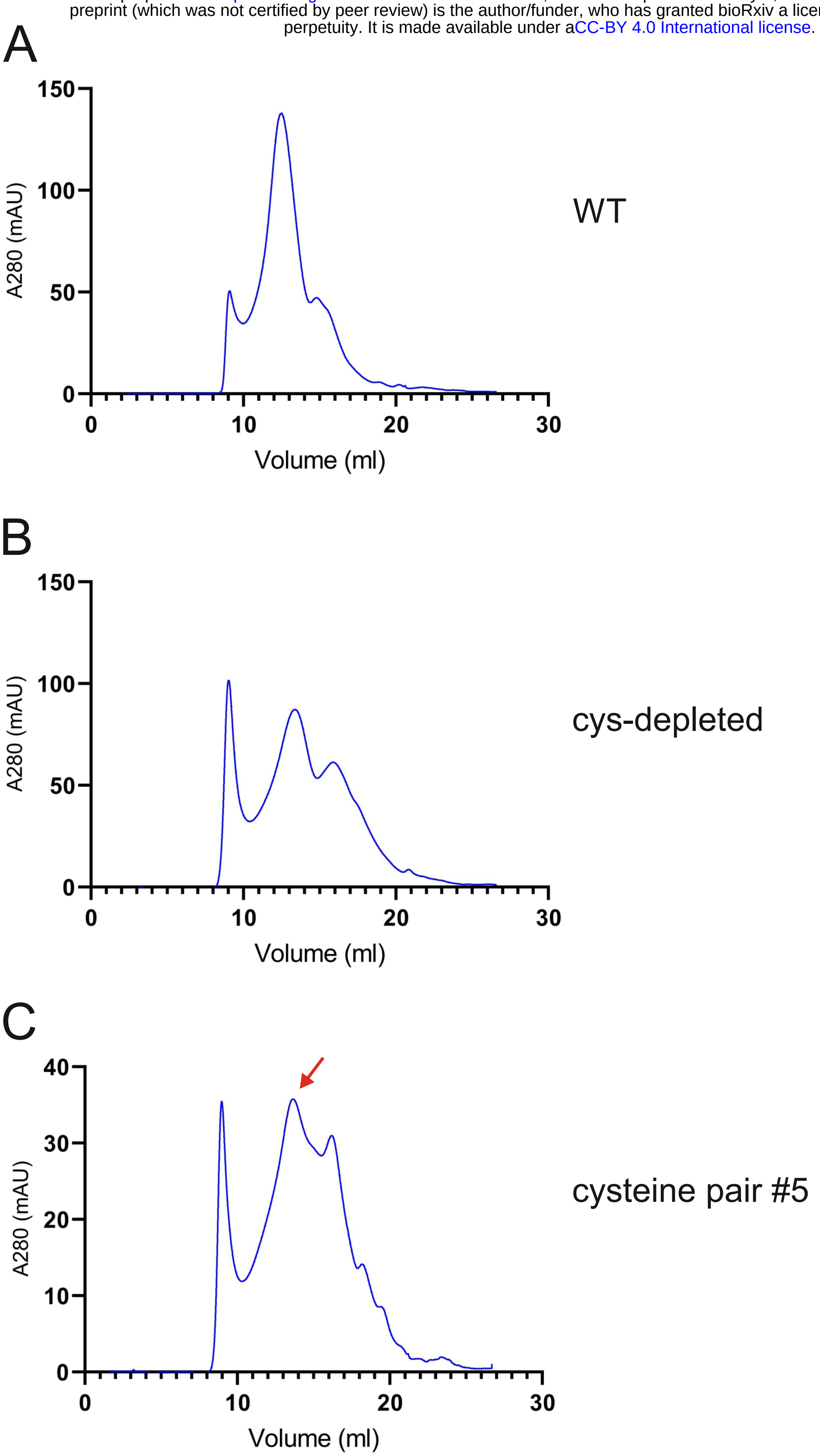

Supplementary Figure 2 | Size exclusion chromatograms. (**A**-**C**) Size exclusion chromatography traces of wild-type MmpS4-MmpL4 (**A**), cys-depleted MmpS4-MmpL4 (**B**) and MmpS4-MmpL4 containing the cysteine pair #5 (**C**). The proteins were separated on a Superose 6 Increase 10/300 GL column. The main peak of MmpS4-MmpL4 containing the cysteine pair #5 eluting at 13.6 ml (indicated by red arrow) was concentrated and analyzed by cryo-EM. The cryo-EM structure of the hexameric $(MmpS4)_3$-$(MmpL4)_3$ complex was determined from this sample.

bioRxiv preprint doi: https://doi.org/10.64898/2026.01.07.698164; this version posted January 8, 2026. The copyright holder for this preprint (which was not certified by peer review) is the author/funder, who has granted bioRxiv a license to display the preprint in perpetuity. It is made available under aCC-BY 4.0 International license.

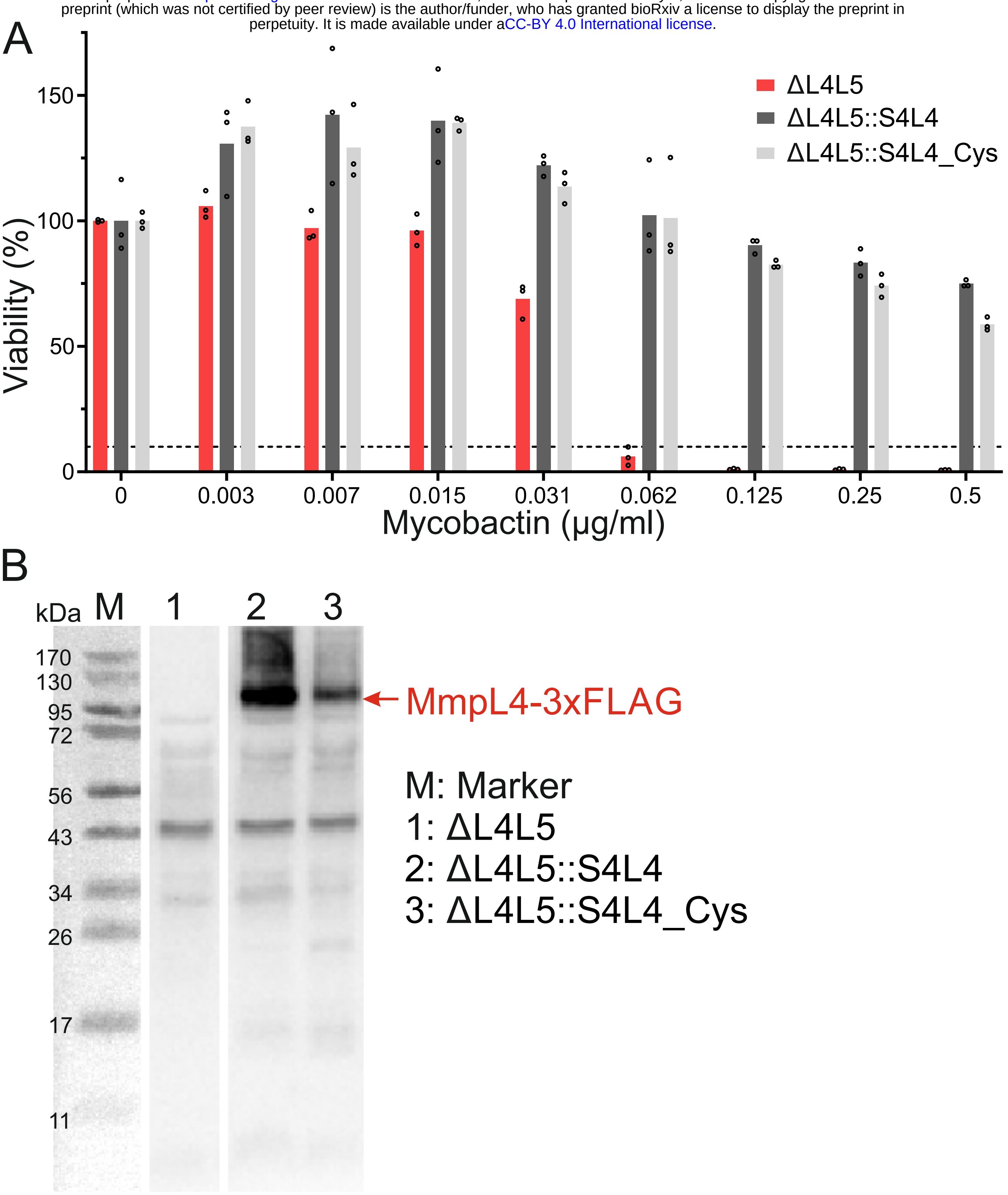

Supplementary Figure 3 | Functional analysis of cysteine depleted mutant. (**A**) Viability of *M. tuberculosis* strains at increasing mycobactin concentrations was determined using the microplate Alamar Blue assay (see methods). Data were normalized to the viability in the absence of mycobactin (100 %). ΔL4L5, *M. tuberculosis* strain lacking *mmpL4* and *mmpL5*; ::S4L4 complementation with *mmpS4-mmpL4*; ::S4L4_Cys complementation with cysteine depleted *mmpS4-mmpL4*. Data points correspond to technical triplicates. (**B**) Western blot analysis of MmpL4 extracted from the strains shown in (**A**) using an anti-3xFLAG antibody (see methods).

bioRxiv preprint doi: https://doi.org/10.64898/2026.01.07.698164; this version posted January 8, 2026. The copyright holder for this preprint (which was not certified by peer review) is the author/funder, who has granted bioRxiv a license to display the preprint in perpetuity. It is made available under aCC-BY 4.0 International license.

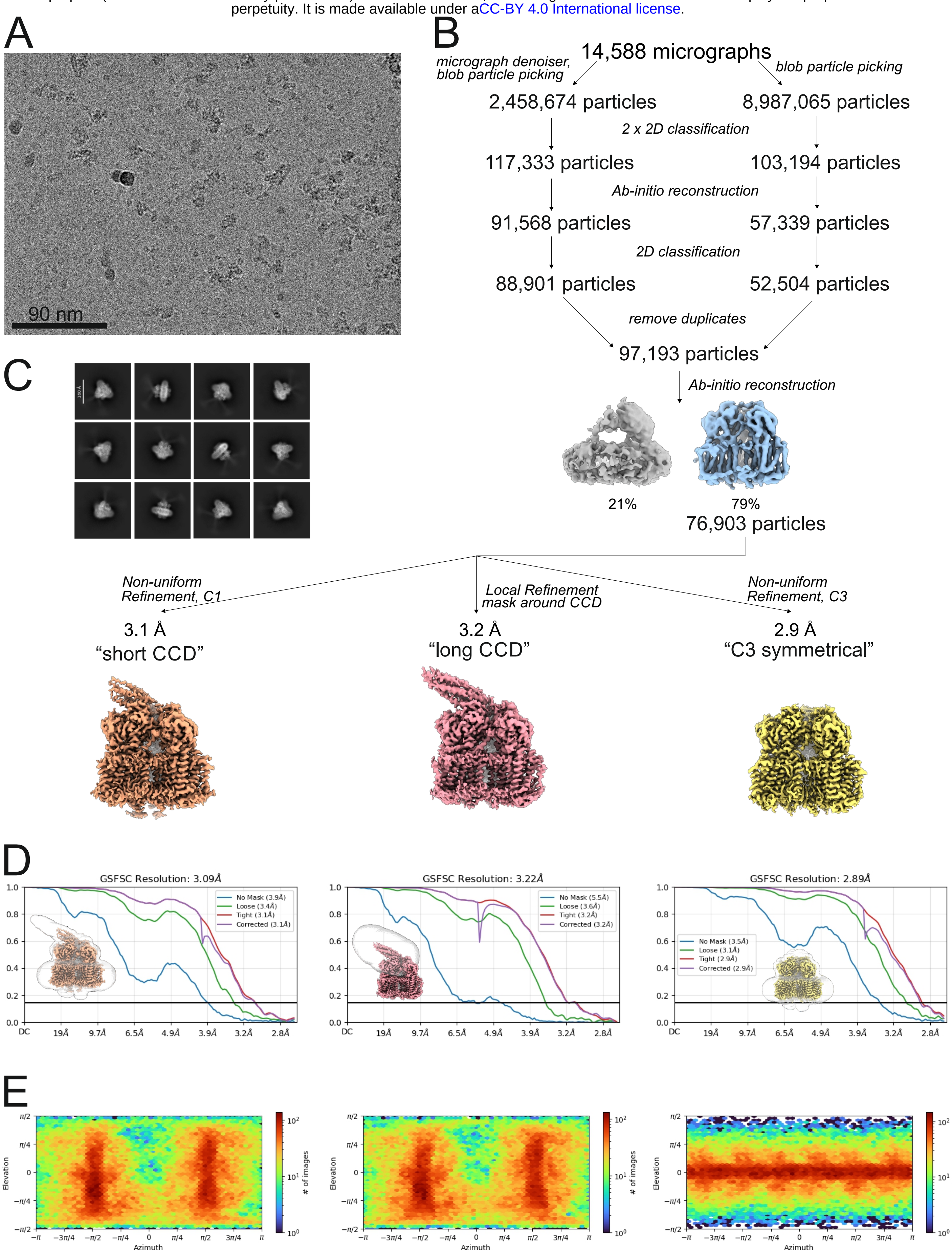

Supplementary Figure 4 | Cryo-EM reconstruction of the hexameric MmpS4-MmpL4 complex. (**A**) Representative cryo-EM micrograph. (**B**) Image processing work flow. (**C**) 2D-class averages. (**D**) FSC plot used for resolution estimation. (**E**) Angular distribution plot of particles included in the final 3D reconstruction.

bioRxiv preprint doi: https://doi.org/10.64898/2026.01.07.698164; this version posted January 8, 2026. The copyright holder for this preprint (which was not certified by peer review) is the author/funder, who has granted bioRxiv a license to display the preprint in perpetuity. It is made available under aCC-BY 4.0 International license.

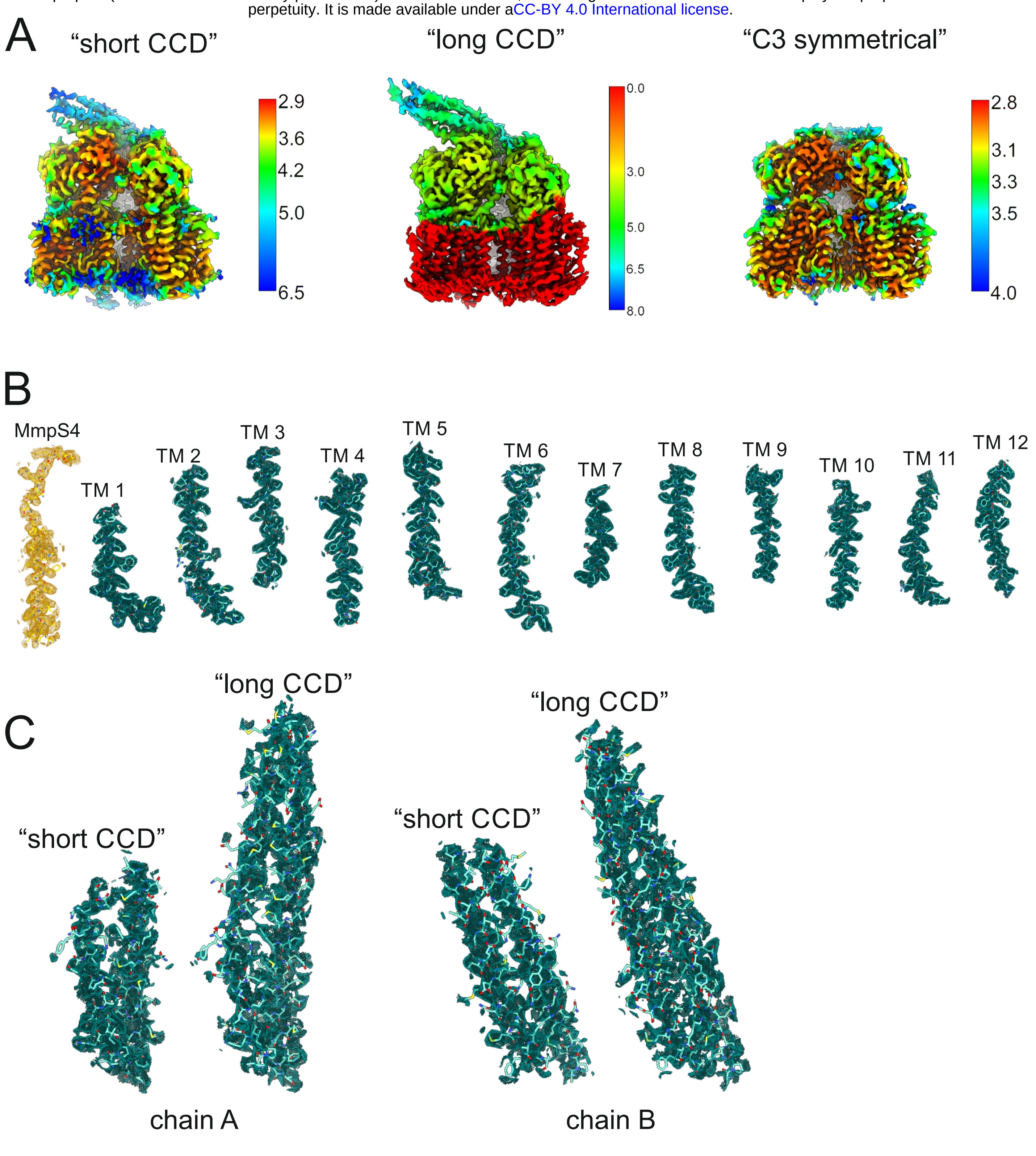

Supplementary Figure 5 | Local resolution estimations and cryo-EM densities of the hexameric MmpS4-MmpL4 complex. (**A**) Final reconstructed maps colored by local resolution. Note that for the "long CCD" map, a large part of the map was not part of the mask and thus no local resolution could be determined (red parts, resolution arbitrarily set to zero). (**B**) Cryo-EM densities of the hexameric MmpS4-MmpL4 complex "short CCD" with the respective refined model superimposed. The model is shown as sticks and structural elements are labelled. Transmembrane helices (TM) are colored in green, MmpS4 in yellow. Densities were contoured at 5.4σ. (**C**) Cryo-EM densities of CCDs from "short CCD" and "long CCD" models, chains A on the left and chains B on the right. Densities were contoured at 4.2σ. Cryo-EM reconstruction of the hexameric MmpS4-MmpL4 complex.

bioRxiv preprint doi: https://doi.org/10.64898/2026.01.07.698164; this version posted January 8, 2026. The copyright holder for this preprint (which was not certified by peer review) is the author/funder, who has granted bioRxiv a license to display the preprint in perpetuity. It is made available under aCC-BY 4.0 International license.

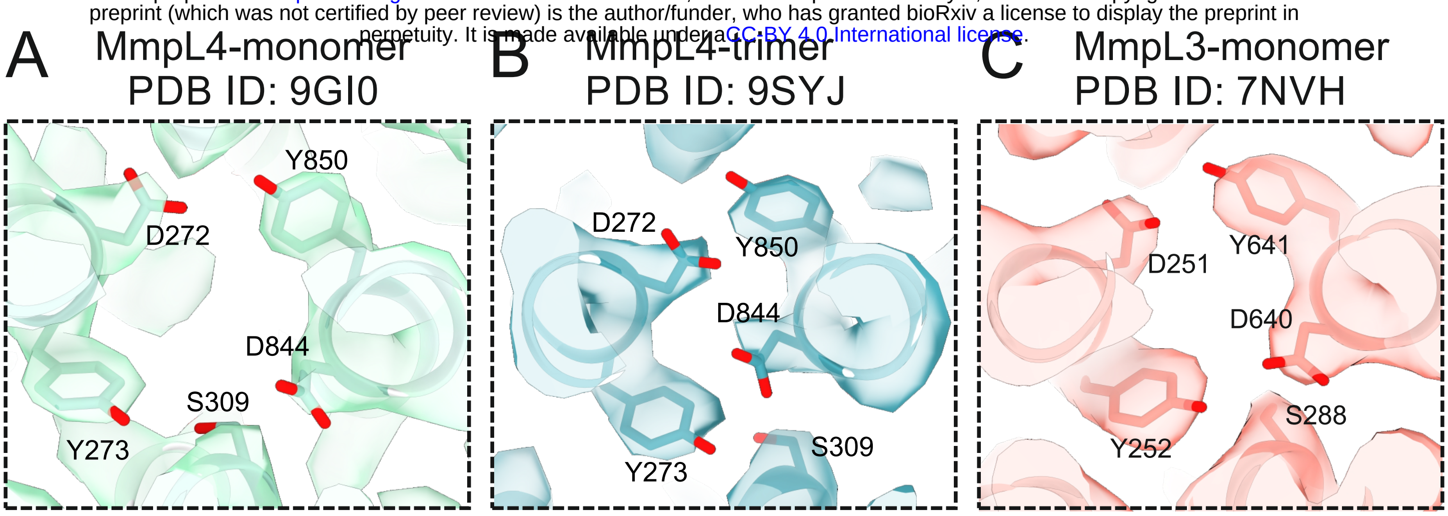

Supplementary Figure 6 | Cryo-EM densities of DY-pairs. The conserved DY-pairs and S309(MmpL4)/S288(MmpL3) are shown as sticks and viewed from the periplasm as in main Fig. 3. (**A**) Monomeric MmpL4 (PDB ID: 9GI0) contoured at 11.9σ. (**B**) Trimeric MmpL4 (this work, PDB ID: 9SYJ, chain A) contoured at 8.9σ. (**C**) Monomeric MmpL3 (PDB ID: 7NVH) contoured at 4.5σ.

bioRxiv preprint doi: https://doi.org/10.64898/2026.01.07.698164; this version posted January 8, 2026. The copyright holder for this preprint (which was not certified by peer review) is the author/funder, who has granted bioRxiv a license to display the preprint in perpetuity. It is made available under aCC-BY 4.0 International license.

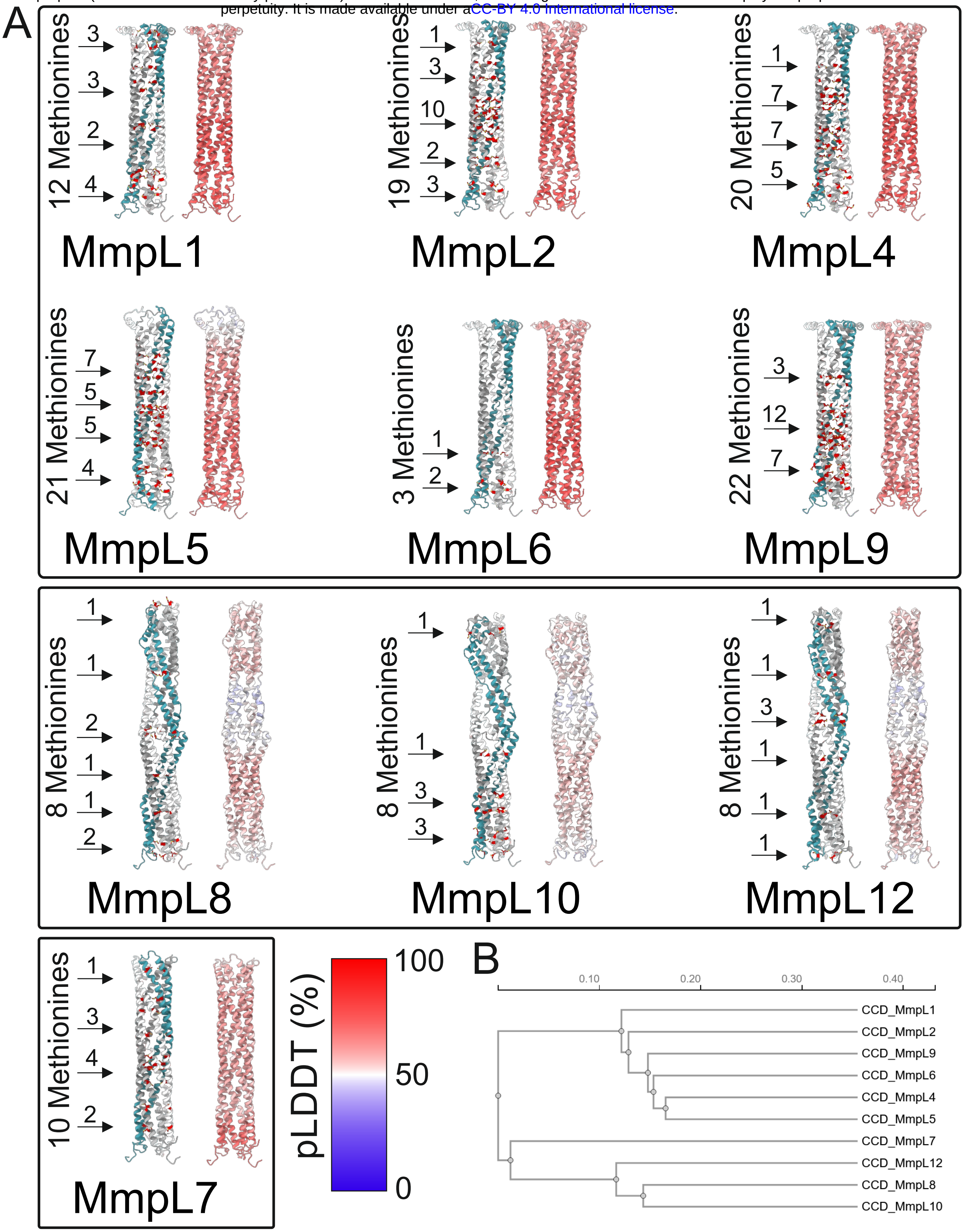

Supplementary Figure 7 | Comparative analysis of CCDs. (**A**) CCDs of *M. tuberculosis* MmpL proteins shown as cartoons. The models are AlphaFold3 predictions of the respective MmpS-MmpL hexamers (in case an MmpS protein is present) or the MmpL trimers. For each CCD, methionine residues are shown as red sticks and the number of methionine residues per α-helical hairpin is provided in the pictures on the left and in the pictures on the right, the models are colored according to their Predicted Local Distance Difference Test (pLDDT) values. The coloring scheme is provided at the bottom. (**B**) Phylogenetic tree of the CCDs shown in (**A**).

bioRxiv preprint doi: https://doi.org/10.64898/2026.01.07.698164; this version posted January 8, 2026. The copyright holder for this preprint (which was not certified by peer review) is the author/funder, who has granted bioRxiv a license to display the preprint in perpetuity. It is made available under aCC-BY 4.0 International license.

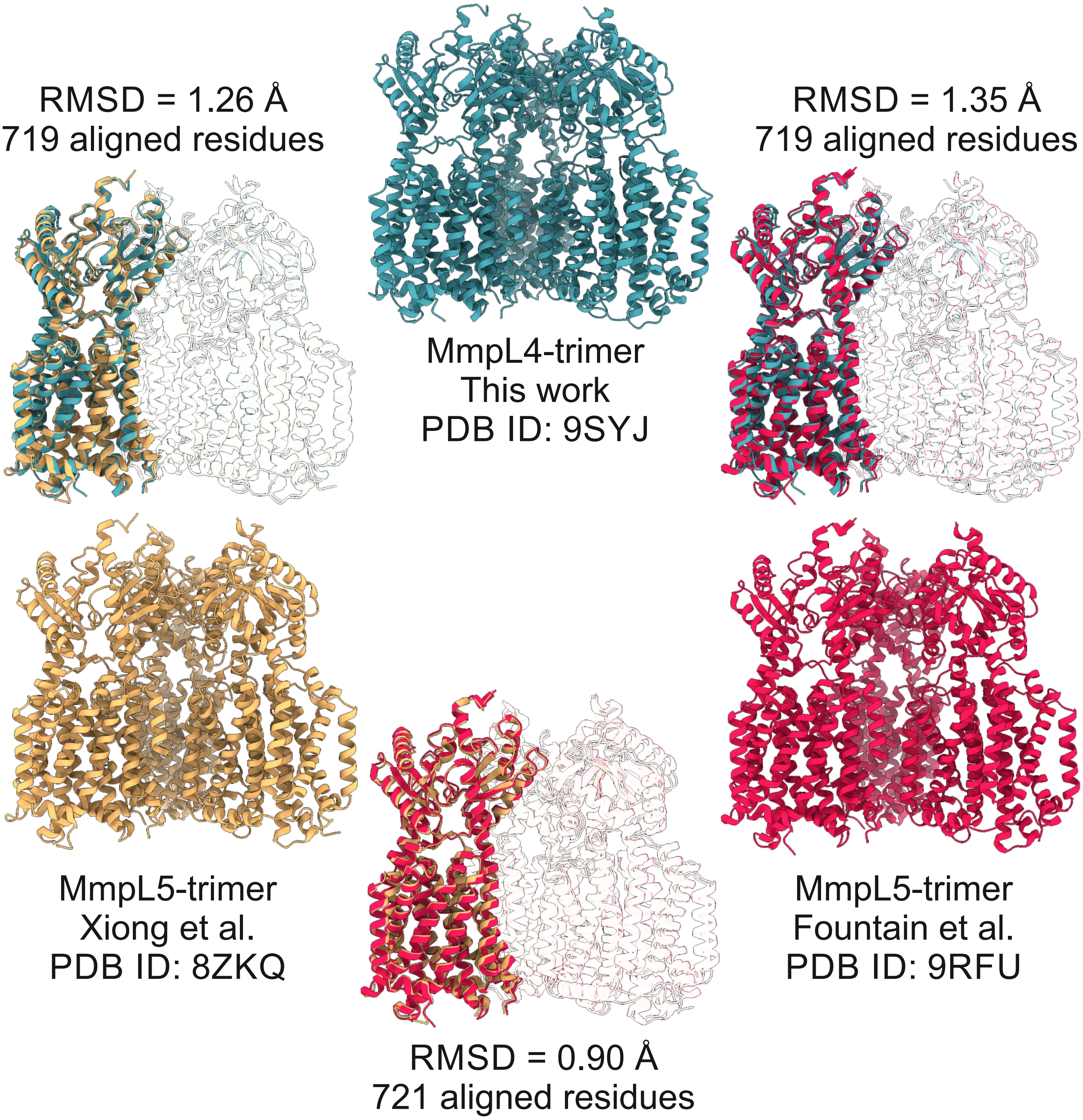

Supplementary Figure 8 | Structural comparisons of trimeric *M. tuberculosis* MmpL4 and MmpL5. The first protomers of the trimeric MmpL4 structure (this study, PDB ID: 9SYJ, chain A) and the two trimeric MmpL5 structures (Xiong et al., PDB ID: 8ZKQ, chain A / Fountain et al., PDB ID: 9RFU, chain D) were superimposed using the matchmaker tool of ChimeraX (Needleman-Wunsch alignment algorithm, BLOSUM-62 similarity matrix). The respective RMDS values are depicted directly in the figure, along with the number of aligned residues.
